# CircFAM73A promotes the cancer stem cell-like properties of gastric cancer through the miR-490-3p/HMGA2 positive feedback loop and HNRNPK-mediated β-catenin stabilization

**DOI:** 10.1186/s13046-021-01896-9

**Published:** 2021-03-17

**Authors:** Yiwen Xia, Jialun Lv, Tianlu Jiang, Bowen Li, Ying Li, Zhongyuan He, Zhe Xuan, Guangli Sun, Sen Wang, Zheng Li, Weizhi Wang, Linjun Wang, Zekuan Xu

**Affiliations:** 1 Department of General Surgery, The First Affiliated Hospital of Nanjing Medical University, No.300, Guangzhou Road, Nanjing, Jiangsu Province China; 2 Jiangsu Key Lab of Cancer Biomarkers, Prevention and Treatment, Collaborative Innovation Center for Cancer Personalized Medicine, Nanjing Medical University, Nanjing, 210029 Jiangsu Province China

## Abstract

**Background:**

Circular RNAs (circRNAs) have emerged as a new subclass of regulatory RNAs that play critical roles in various cancers. Cancer stem cells (CSCs), a small subset of cancer cells, are believed to possess the capacities to initiate tumorigenesis and promote progression. Although accumulating evidence has suggested that cells with CSC-like properties are crucial for the malignancy of gastric cancer (GC), it remains unclear whether circRNAs are related to the acquisition of CSC-like properties in GC.

**Methods:**

CircFAM73A expression was analyzed by GEO datasets and verified in GC samples. The roles of circFAM73A in GC cell proliferation, migration, cisplatin resistance, and CSC-like properties were determined by a series of functional experiments both in vitro and in vivo. RNA pulldown was used to explore the miRNAs and proteins binding to circFAM73A. Bioinformatic analysis and experimental verification confirmed the downstream targets of circFAM73A. The regulation of circFAM73A by HMGA2 was verified by ChIP and RIP assays.

**Results:**

Elevated circFAM73A expression was confirmed in GC tissues, and higher circFAM73A predicted poor prognosis in GC patients. The upregulation of circFAM73A enhanced CSC-like properties in GC, thus facilitating cell proliferation, migration, and cisplatin resistance. Mechanistically, circFAM73A promoted GC malignancy by regulating miR-490-3p/HMGA2 in a positive feedback loop and recruiting HNRNPK to facilitate β-catenin stabilization. Moreover, HMGA2 further enhanced E2F1 and HNRNPL activity, which in turn promoted circFAM73A expression.

**Conclusions:**

Our work demonstrates the crucial role of circFAM73A in the CSC-like properties of GC and uncovers a positive feedback loop in circFAM73A regulation that leads to the progression of gastric cancer, which may provide new insights into circRNA-based diagnostic and therapeutic strategies.

**Supplementary Information:**

The online version contains supplementary material available at 10.1186/s13046-021-01896-9.

## Background

Gastric cancer (GC) remains one of the most frequently diagnosed cancers and is responsible for over 1,000,000 new cases and approximately 783,000 deaths annually, making it the fifth most common cancer and ranking as the third leading cause of cancer-associated mortality worldwide [1]. Therefore, novel effective diagnostic and therapeutic strategies are urgently needed.

Cancer stem cells (CSCs) are defined as the fraction of cells that retain the capacities of self-renewal and differentiation [2]. Although CSCs only account for a small proportion of cancer cells, they exhibit prominent features, including tumorigenesis, chemotherapy resistance, and high metastatic potential, which have been shown to be largely responsible for cancer metastasis, recurrence, and treatment failure [3, 4]. Emerging evidence has supported the existence of CSCs in GC, where they were isolated based on the expression of CSC-specific surface markers [5, 6]. Nevertheless, the underlying regulatory mechanism of CSCs in GC remains to be elucidated.

Circular RNA (circRNA) is a widespread special form of RNA that is mostly generated from backsplicing of precursor mRNA (pre-mRNA) [7, 8]. Although the identification of circular RNA dates back to the mid-1970s [9], these transcripts have largely been assumed to be aberrant splicing byproducts and thus remain poorly researched [10, 11]. With the rapid growth of next-generation profiling of noncoding RNAs, however, the simplistic view of circRNAs as transcriptional noise has given way to a deeper understanding of their various biological functions. CircRNAs are now speculated to be highly conserved and stable, as circular structures resist most RNA decay mechanisms [12, 13]. Mounting evidence indicates the involvement of circRNAs in several physiological and pathological processes [14–16], including cancer [17–20]. Therefore, circRNAs may also serve as potential biomarkers in many malignancies [17, 21, 22]. However, little is known about the overall pathophysiological effects of circRNAs on stem cell-like properties.

Here, we investigated whether circRNAs are involved in CSC-like properties in GC. Our findings demonstrated that circFAM73A promotes gastric cancer stem cell-like properties, thus promoting cell malignancy in GC. More importantly, elevated circFAM73A predicts poor prognosis in GC patients. CircFAM73A regulates HMGA2 expression by absorbing miR-490-3p, while HMGA2 facilitates the transcriptional activation of FAM73A by E2F1 and enhances the efficiency of circFAM73A circularization by HNRNPL, generating a positive feedback loop to further elevate circFAM73A expression. In addition, circFAM73A recruits HNRNPK, enhances the interaction between HNRNPK and β-catenin and facilitates the stability of β-catenin.

Hence, our study uncovers a novel mechanism of circFAM73A that promotes GC progression and demonstrates the intrinsic value of circFAM73A as a prognostic predictor and potential therapeutic target.

## Methods

### Clinical samples and cell lines

Primary GC samples in this study were obtained from 100 patients who underwent radical resection for GC in the Department of Gastric Surgery, the First Affiliated Hospital of Nanjing Medical University between 2015 and 2016. No patient received adjuvant chemotherapy before surgery. This study was approved by the Ethics Committee of the First Affiliated Hospital of Nanjing Medical University, and all participants provided written informed consent.

All human GC cells, including AGS, HGC27, MKN45, MGC803, SGC7901, and BGC823 cells, and the normal human gastric epithelial cell line GES-1 were purchased from Shanghai Institutes for Biological Sciences. All cell lines were cultured in RPMI-1640 (Gibco, USA) supplemented with 10% fetal bovine serum (WISENT, Canada) and antibiotics (1% penicillin/streptomycin; Gibco) and incubated in a humidified cell chamber (5% CO_2_, 37 °C).

### RNA extraction and quantitative real-time polymerase chain reaction

Total RNA was extracted by TRIzol reagent (Invitrogen, Carlsbad, CA, USA), and the nuclear and cytoplasmic fractions were isolated by NE-PER Nuclear and Cytoplasmic Extraction Reagents (Thermo Scientific) according to the manufacturer’s protocols. The concentration and quality of isolated RNA were measured by a NanoDrop spectrophotometer (ND-100, Thermo). Reverse transcription of miRNA was performed using a New Poly(A) Tailing Kit (Thermo Fisher Scientific). For circRNA and mRNA, total RNA was reverse transcribed to cDNA by a PrimeScript RT Master Mix Kit (TaKaRa, RR036A, Japan). qRT-PCR was conducted using Universal SYBR Green Master Mix (4913914001, Roche) with a 7500 Real-Time PCR System (Applied Biosystems, USA). The levels of miRNA were normalized to that of small nuclear U6, and *GAPDH* was used as an internal control for the relative expression of circRNAs and mRNAs. The relative expression levels were calculated using the 2^−ΔΔCT^ method, and the primers used are listed in Supplementary Table S1.

### Actinomycin D and RNase R treatment

BGC823 and SGC7901 cells were seeded in a 24-well plate overnight. Total RNA was extracted after treatment with actinomycin D (2 mg/ml) or DMSO (Sigma-Aldrich, USA) for different periods of time (0, 6, 12, 18, and 24 h). Total RNA (5 μg) was incubated with or without 20 U RNase R (Geneseed, China) at 37 °C for 20 min. After treatment with actinomycin D or RNase R, the stability of circFAM73A and FAM73A mRNA was measured by qRT-PCR.

### Western blot and immunoprecipitation

Total protein was extracted with RIPA lysis buffer. Protein concentrations were measured by a BCA protein assay kit (Beyotime Biotechnology, Jiangsu, China). Cell lysates were separated on SDS-polyacrylamide gels and then transferred onto polyvinylidene difluoride (PVDF) membranes. After the membranes were blocked in 5% skim powdered milk for 2 h, they were incubated with primary antibodies overnight at 4 °C and with HRP-conjugated secondary antibodies for 2 h at room temperature. The blots were visualized by ECL chemiluminescent reagent (Millipore, MA, USA). The antibodies used in this study are listed in Supplementary Table S2.

For immunoprecipitation, GC cells were washed with 4 °C PBS followed by lysis in lysis buffer. After preclearance with Protein A/G PLUS-Agarose (SC-2003, Santa Cruz), cell lysates were immunoprecipitated with the indicated antibodies. Purified immunoglobulin G (IgG) from host species was used as a control. Protein A/G PLUS-Agarose was subsequently used to capture the immunocomplexes. Immunoprecipitated proteins were eluted by boiling in SDS-PAGE loading buffer and analyzed by Western blots.

### Transfection

The human circFAM73A expression vector and si-circFAM73A expression vector were purchased from GeneChem (Shanghai, China). MiR-490-3p mimics and inhibitors were synthesized by GenePharma (Shanghai, China). The transfection procedure was carried out with Lipofectamine 3000 (Invitrogen) in accordance with the manufacturer’s instructions. For stable transfection, the lentiviruses were constructed by GeneChem (Shanghai, China) and transfected according to the manufacturer’s protocols.

### Cell counting Kit-8 and colony formation assays

The Cell Counting Kit-8 (CCK-8) (Dojindo, Japan) method was used to investigate cell proliferation. Cells were seeded in 96-well plates at a density of 1500 cells per well. The optical density (OD) values at 450 nm of each well were measured every 24 h for 5 times.

To asscess cell viability, we seeded 5000 cells into 96-well plates per well. After 24 h, the cells were cultured with medium containing various cisplatin concentrations for another 48 h. Cell viability was quantified by CCK-8 kits.

For the colony formation assay, cells were seeded in 6-well plates at a density of 500 cells per well. After 2 weeks, the colonies were fixed in methanol for 10 min and then stained with 1% crystal violet for another 20 min at room temperature. Images were captured, and cell colonies were counted and analyzed.

### EdU (5-ethynyl-20-deoxyuridine) incorporation assay

The EdU assay was carried out using a Cell-Light EdU DNA Cell Proliferation Kit (RiboBio, Guangzhou, China) according to the manufacturer’s protocols. Briefly, cells were seeded in 96-well plates and incubated with EdU (50 μM) for 2 h. After fixation in 4% paraformaldehyde, the cells were stained with Apollo Dye Solution. Hoechst 33342 was used for the staining of the cell nucleus. Images were captured by an Olympus microscope (Olympus, Tokyo, Japan) for five random fields, and the percentage of EdU-positive cells was determined.

### Flow cytometry

For the cell cycle assay, cells were collected and fixed in ice-cold 75% ethanol overnight. The cells were then incubated with 500 μL of propidium iodide (PI) staining solution for 30 min using a Cycletest Plus DNA Reagent Kit (BD Biosciences). The cell distribution was detected by flow cytometry.

For apoptosis analysis, cells were stained with an Annexin V-FITC/Propidium Iodide (PI) Apoptosis Detection Kit (BD, Biosciences #556547) and detected by flow cytometry.

For detection of the CD44 proportion, cells were resuspended in PBS containing 2% FBS and incubated with FITC-conjugated CD44 (BD Biosciences, 555478) for 20 min. After incubation, the cells were washed and resuspended in 300 μl of PBS and analyzed using flow cytometry. FITC Mouse IgG2b κ Isotype Control (BD Biosciences, 556655) was used as a control.

### Sphere formation assays

Cells were seeded at a density of 5000 cells/well into 6-well ultralow attachment plates (Corning, NY). Fresh stem cell medium (1.5 ml) was added every 2 days. The stem cell medium included DMEM/F12 medium (Invitrogen, Grand Island, NY) supplemented with EGF (20 ng/ml; Invitrogen), bFGF (10 ng/ml; Invitrogen) and 2% B27 (Invitrogen, CA, USA). Ten days after being planted, spheres with diameters > 50 μm were counted and analyzed.

### Organoid culture

Sterile gastric cancer tissues obtained from patients after gastric surgery were cut into small pieces and digested with collagenase A at room temperature for 40 min. Then, cells were resuspended in Matrigel (R&D Systems, Minneapolis, MN, USA). Fifty microliters of Matrigel containing cells with growth factors was seeded in a 24-well plate and supplemented with 500 μl of Organoid Growth Medium (human) (StemCell Technologies, Vancouver, Canada) for organoid growth. Photographs of human GC organoids were taken daily by microscopy.

### Biotinylated RNA pulldown assay

The biotin-coupled cricFAM73A probe, control oligo probe, miR-490-3p wild-type probe, and mutated-type probe were synthesized by RiboBio (Guangzhou, China). Cells were fixed by 1% formaldehyde for 10 min and lysed with lysis buffer containing 25 mM Tris-HCl pH 7.4, 150 mM NaCl, 1 mM EDTA, 1% NP-40, 5% glycerol, 1 U/μL SUPERase-In RNase Inhibitor (Ambion), and Protease Inhibitor Cocktail (Halt). The lysates were then centrifuged at 10,000 g for 10 min. Fifty microliter of the supernatant was saved for input analysis and the remaining part was transferred to a fresh tube. The biotin-coupled RNA complex was pulled down by incubating streptavidin-coated magnetic beads (Invitrogen, Carlsbad, USA) with cell lysates overnight at 4 °C. On the next day, the beads-probes-RNA mixture was washed and the RNA bound to the beads was extracted by TRIzol for subsequent qRT-PCR analysis.

The relative abundance of RNA bound to the probe was analyzed using 2^-ΔCt^ method relative to the RNA levels of input. The miRNA expression bound to circFMA73A was then calculated by the expression of miRNA captured by the circFAM73A probe relative to miRNA captured by the Oligo probe.

### Luciferase reporter assay

A luciferase reporter vector containing the wild-type sequences of circFMA73A or the 3′-UTR of *HMGA2* was constructed, and the mutated constructs were constructed according to the binding sites. Luciferase reporter vectors were then cotransfected with miR-490-3p mimic or control vectors. Forty-eight hours after transfection, the relative luciferase activity was calculated as the ratio between firefly and Renilla luciferase activities detected by a dual-luciferase system (Promega, Madison, WI).

### Chromatin immunoprecipitation assay

**A** Pierce™ Magnetic ChIP Kit (26157, Thermo Fisher Scientific) was applied for the chromatin immunoprecipitation (ChIP) assay. In brief, BGC823 and SGC7901 cells were fixed by adding 37% formaldehyde to a final concentration of 1% and then terminated by glycine solution. Cells were then resuspended in lysis buffer. Cell lysates were sonicated on ice to shear crosslinked DNA to lengths between 100 and 1000 bp for sufficient pulldown efficiency. The cell lysates were precleared with protein A/G magnetic beads before incubation with E2F1 antibody (3742, Cell Signaling Technology) or control antibody normal rabbit IgG (12–370, Sigma-Aldrich) overnight at 4 °C. After incubation with antibodies, protein A/G magnetic beads were added to capture the antibody/histone/DNA complex. Before DNA extraction, 1/5 of the cell lysate was removed for Western blot analysis. The DNA was then extracted, and the target DNA was detected by qRT-PCR. The primers designed for the ChIP assay according to the potential E2F1 binding sites in the FAM73A promoter region are listed in Supplementary Table S1.

### RNA-binding protein immunoprecipitation assay

RNA-binding protein immunoprecipitation assays were performed using the Magna RIP™ RNA-Binding Protein Immunoprecipitation Kit (17–700, Merck Millipore) according to the manufacturer’s protocols. Briefly, BGC823 and SGC7901 cells in plates were crosslinked with 1% formaldehyde after washing with 4 °C PBS and then lysed in lysis buffer. Cell lysates were then sonicated on ice. HNRNPL antibody (65043, Cell Signaling Technology) or control antibody normal rabbit IgG (12–370, Sigma-Aldrich) was incubated with washed magnetic beads for 30 min at room temperature. The bead-antibody complexes were then incubated with cell lysates overnight at 4 °C. The RNA was extracted and measured by qRT-PCR. The primers designed for the RIP assay according to the potential HNRNPL binding sites within flanking introns of circFAM73A are listed in Supplementary Table S1.

### RNA fluorescence in situ hybridization (FISH)

Fam-labeled circFAM73A and Cy3-labeled miR-490-3p probes were designed and synthesized by Servicebio (Wuhan, China). FISH experiments were performed according to the manufacturer’s protocols. Briefly, for the cell assay, cells were fixed in 4% paraformaldehyde and permeabilized with 0.1% Triton X-100. For GC tissues, 4-mm thick sections were cut from paraffin-embedded blocks and then deparaffinized and rehydrated. Hybridization of cells or tissue was performed with specific probes in a dark moist chamber at 37 °C overnight. The slices were sealed with parafilm containing DAPI. Images were acquired by a Leica SP5 confocal microscope system (Leica Microsystems, Mannheim, Germany).

### Immunohistochemistry (IHC) staining

All specimens were fixed in 4% formalin, embedded in paraffin and sectioned into 5 μm sections. The 5 μm sections were incubated with primary antibodies (HMGA2, Proteintech 20,795–1-AP; CD44, Abcam ab157107) overnight at 4 °C, followed by incubation with secondary antibodies at room temperature for 30 min. Next, sections were stained with DAB solution for 5 min. The IHC staining was scored by the percentage of positive cells (graded as 0, < 5%; 1, 5–25%; 2, 26–50%; 3, 51–75%; and 4, > 75%) and the intensity of cell staining (graded as 0, no staining; 1, weak; 2, moderate; and 3, strong).

### Animal experiment

Four -week-old female BALB/c nude mice were purchased from the Department of Laboratory Animal Center of Nanjing Medical University. All animal studies were approved by the Nanjing Medical University Ethics Committee.

For the xenograft tumor growth assay, 1 × 10^6^ transfected cells suspended in 100 μL were subcutaneously injected into the axilla of nude mice. The width and length of the tumors were measured once a week, and the tumor volumes were calculated as (width^2^ × length)/2. Four weeks after injection, the mice were sacrificed, and tumor weights were measured.

For the lung metastasis model, transfected luciferase-labeled cells were injected into the caudal veins of mice, and metastasis was monitored using an in vivo imaging system (IVIS). Six weeks after injection, the mice were sacrificed, and lung tissues were harvested for hematoxylin-eosin staining.

For the liver metastasis model, cells were injected into the portal veins of mice, and 6 weeks after injection, the mice were sacrificed, and liver tissues were harvested for hematoxylin-eosin staining.

### Statistical analysis

Statistical analyses were conducted by SPSS 20.0 (IBM, SPSS, IL, USA) and GraphPad Prism. All data are presented as the mean ± standard deviation (S.D.). Student’s t-test was performed to assess statistical significance between two groups. Pearson’s correlation analysis was used to analyze the association between circFAM73A expression and clinicopathologic parameters. Overall survival (OS) was analyzed by the Kaplan-Meier method and log-rank test. Univariate analysis and multivariate models were performed with Cox proportional hazards regression models.

## Results

### CircFAM73A is upregulated in GC, and high circFAM73A expression predicts poor prognosis

**Fig. 1:**
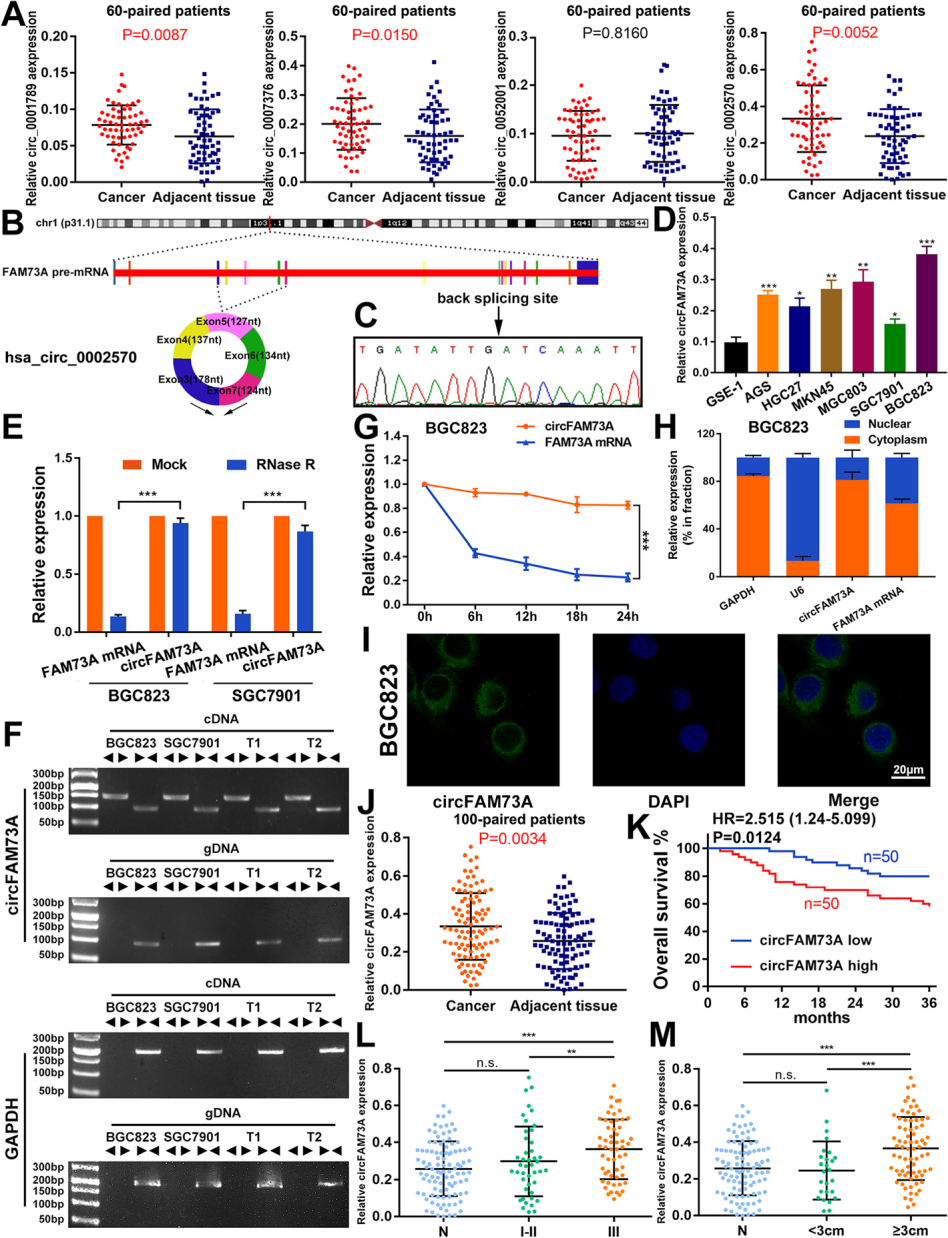
CircFAM73A is upregulated in GC and high circFAM73A predicts poor prognosis. **a** Validated the expression of hsa_circ_0001789, hsa_circ_0007376, hsa_circ_0052001 and hsa_circ_0002570 in the 60 paired GC and adjacent tissues by qRT-PCR. **b** Schematic drawing illustrating circFAM73A (hsa_circ_0002570) (700 bp) arose from exon 3,4,5,6,7 of the FAM73A gene. **c** Arrow represents the “head-to-tail” splicing sites of circFAM73A confirmed by Sanger sequencing. **d** Relative circFAM73A expression in 6 GC cell lines relative to normal human gastric epithelial cell line GES-1. **e** qRT-PCR analysis of the level of circFAM73A and linear FAM73A mRNA after treatment with RNase R in BGC823 and SGC7901 (normalized to mock treatment). **f** The existence of circFAM73A was validated in BGC823 and SGC7901 and two GC samples (T1 and T2) by RT-PCR. Divergent primers amplified circFAM73A from cDNA, but not from genomic DNA (gDNA). GAPDH was used as a negative control. **g** Relative levels of circFAM73A and FAM73A mRNA were measured by qRT-PCR in BGC823 treated with Actinomycin D for different periods of time. **h** Relative levels of GAPDH (positive control for cytoplasmic fraction), U6 (positive control for nuclear fraction), circFAM73A, and FAM73A mRNA from nuclear and cytoplasmic fractions in BGC823. **i** Fluorescence in situ hybridization (FISH) was conducted to determine the subcellular localization of circFAM73A in BGC823. DAPI was used for nuclei staining. Scale bar: 20 μm. **j** Relative circFAM73A expression in additional 100 paired GC and adjacent tissues. **k** Overall survival analysis based on circFAM73A expression in 100 GC patients. The median circFAM73A expression is used as a cutoff. **l** The association of circFAM73A expression and TMN stage through qRT-PCR. **m** The association of circFAM73A expression and tumor size through qRT-PCR. Graph represents mean ± SD; *p < 0.05, **p < 0.01, and ***p < 0.001

To assess the circRNAs involved in GC, we searched the circRNA datasets established for gastric cancer in the GEO datasets. Six datasets were found, and GSE83521 was chosen for the subsequent analysis, as it contains the highest number of samples. Several differentially expressed circRNAs were assessed and then filtered according to their log fold change ≥2 and adjusted *p* value ≤0.01. Four circRNAs, hsa_circ_0001789, hsa_circ_0007376, hsa_circ_0052001, and hsa_circ_0002570, were screened out. Next, we verified their expression by qRT-PCR analysis in our 60 paired cancer tissues and their matched adjacent noncancer tissues from GC patients. As shown in Fig. 1a, the most significant expression change was observed for hsa_circ_0002570, thus prompting us to further investigate its role in GC malignancy.

Hsa_circ_0002570 (circFAM73A) originates from exons 3, 4, 5, 6, and 7 of the FAM73A gene (UCSC data in NCBI) (Fig. 1b). We validated the head-to-tail back splicing in the RT–PCR product of circFAM73A using Sanger sequencing (Fig. 1c). qRT-PCR results showed the highest circFAM73A abundance in BGC823 cells and the lowest in SGC7901 cells (Fig. 1d). We therefore selected BGC823 and SGC7901 cell lines for subsequent studies. As shown in Fig. 1e, circFAM73A was resistant to digestion by RNase R exonuclease compared to the linear form of FAM73A in BGC823 and SGC7901 cells. To avoid the possibility of trans-splicing or genomic rearrangements, we conducted several universal circRNA detection experiments [23]. We first designed divergent primers to amplify circFAM73A and convergent primers to amplify FAM73A mRNA. Using cDNA (complementary DNA) and gDNA (genomic DNA) from BGC823 and SGC7901 cells and two random GC tissues as templates, we found that circFAM73A was only amplified from cDNA by divergent primers, whereas no amplification product was obtained when using gDNA (Fig. 1f). Actinomycin D, an transcriptional inhibitor, was then used to measure the half-life of circFAM73A and FAM73A in BGC823 and SGC7901 cells. The results indicated that circFAM73A was more stable than FAM73A mRNA (Fig. 1g and S1a). These findings clearly demonstrated the circular characteristics of circFAM73A.

Next, we examined the relative expression levels of circFAM73A in the cytoplasm and nuclear compartments of BGC823 and SGC7901 cells. qRT-PCR demonstrated that circFAM73A preferentially localized in the cytoplasm (Fig. 1h and S1b), which was confirmed using fluorescence in situ hybridization (FISH) against circFAM73A (Fig. 1i and S1c).

High expression of circFAM73A was then authenticated in an additional 100 paired GC tissue samples (Fig. 1j) using qRT-PCR. Sixty-three percent of the samples (*n* = 63) exhibited higher expression levels in the cancer tissue samples than in the matched noncancerous tissue samples (Fig. S1d and S1e). Our analysis of the clinicopathological characteristics showed that circFAM73A expression significantly correlated with TNM stage and tumor size (Table 1). The expression of circFAM73A in tumor tissues at stage III was notably higher than that at stage I-II and in matched adjacent tissues, whereas the abundance in tumors at stage I-II showed no difference from that in the noncancerous tissues (Fig. 1l). Similarly, we also found that the expression in tumors larger than 3 cm was considerably higher than that in tumors smaller than 3 cm and in adjacent tissues (Fig. 1m). In contrast, other clinicopathological characteristics, including age, sex, tumor site, lymph node metastasis and blood vessel invasion (Fig. S1f-1j ), showed no correlation. Moreover, GC patients with higher circFAM73A expression had significantly shorter overall survival than those with lower circFAM73A expression by Kaplan–Meier survival analysis (Fig. 1k). Further Cox multivariate survival analysis revealed high circFAM73A expression as an independent prognostic factor for poor survival of GC patients (hazard ratio [HR] = 2.171, 95% confidence interval [CI] = 1.015–4.645, *p* = 0.046) (Table 2). In contrast, no obvious change in linear FAM73A mRNA was found in our GC samples (Fig. S1k), and FAM73A showed no correlation with the prognosis of GC patients both in our samples and TCGA database (Fig. S1l and S1m).

**Table 1:** Correlation between circFAM73A expression and the clinicopathologic parameter of 100 GC patients

Clinicopathologic parameter	Number	Number of patients		***p*** value
		circFAM73A^**low**^	circFAM73A^**high**^	
**Age**				
< 60 years	36	16	20	0.532
≥ 60 years	64	34	30	
**Gender**				
Male	75	39	36	0.645
Female	25	11	14	
**Tumor size**				
< 3 cm	26	19	7	0.011*
≥ 3 cm	74	31	43	
**Tumor site**				
Proximal	44	24	20	0.546
Non-proximal	56	26	30	
**Lymph node metastasis**				
N0	38	24	14	0.063
N1-N3	62	26	36	
**TNM stage**				
I-II	44	28	16	0.026*
III	56	22	34	
**Blood vessel invasion**				
Negative	73	40	33	0.176
Positive	27	10	17	

* *p* < 0.05

**Table 2:** Univariate and multivariate cox regression analysis of overall survival in 100 GC patients

Clinicopathologic parameter	Overall survival			
	Univariate analysis		Multivariate analysis	
	HR (95% CI)	***p*** value	HR (95% CI)	***p*** value
Age (≥60 years vs < 60 years)	0.850 (0.412–1.751)	0.659		
Gender (female vs male)	1.659 (0.781–3.524)	0.188		
Tumor size (≥3 cm vs < 3 cm)	1.292 (0.557–2.999)	0.551		
Tumor site (proximal vs non-proximal)	1.284 (0.623–2.646)	0.497		
Lymph node metastasis (N1-N3 vs N0)	3.196 (1.310–7.799)	0.011*	2.806 (1.140–6.902)	0.025*
TNM stage (III vs I-II)	2.860 (1.278–6.402)	0.011*		
Blood vessel invasion (positive vs negative)	2.004 (0.972–4.132)	0.060		
circFAM73A expression (high vs low)	2.518 (1.185–5.349)	0.016*	2.171 (1.015–4.645)	0.046*

* *p* < 0.05

Taken together, these data proved the upregulation of circFAM73A in GC and its clinical significance in GC patients.

### CircFAM73A promotes the proliferation and migration and facilitates cisplatin resistance of GC in vitro

To elucidate the biological function of circFAM73A, we designed two siRNAs to specifically target the backsplice junction (Fig. S2a). Si-circ-1 successfully suppressed circFAM73A expression with no effects on the levels of linear FAM73A in BGC823 and SGC7901 cells (Fig. S2b). In addition, we transfected overexpression vectors into both cell lines, and the efficiency was verified via qRT-PCR (Fig. S2c).

**Fig. 2:**
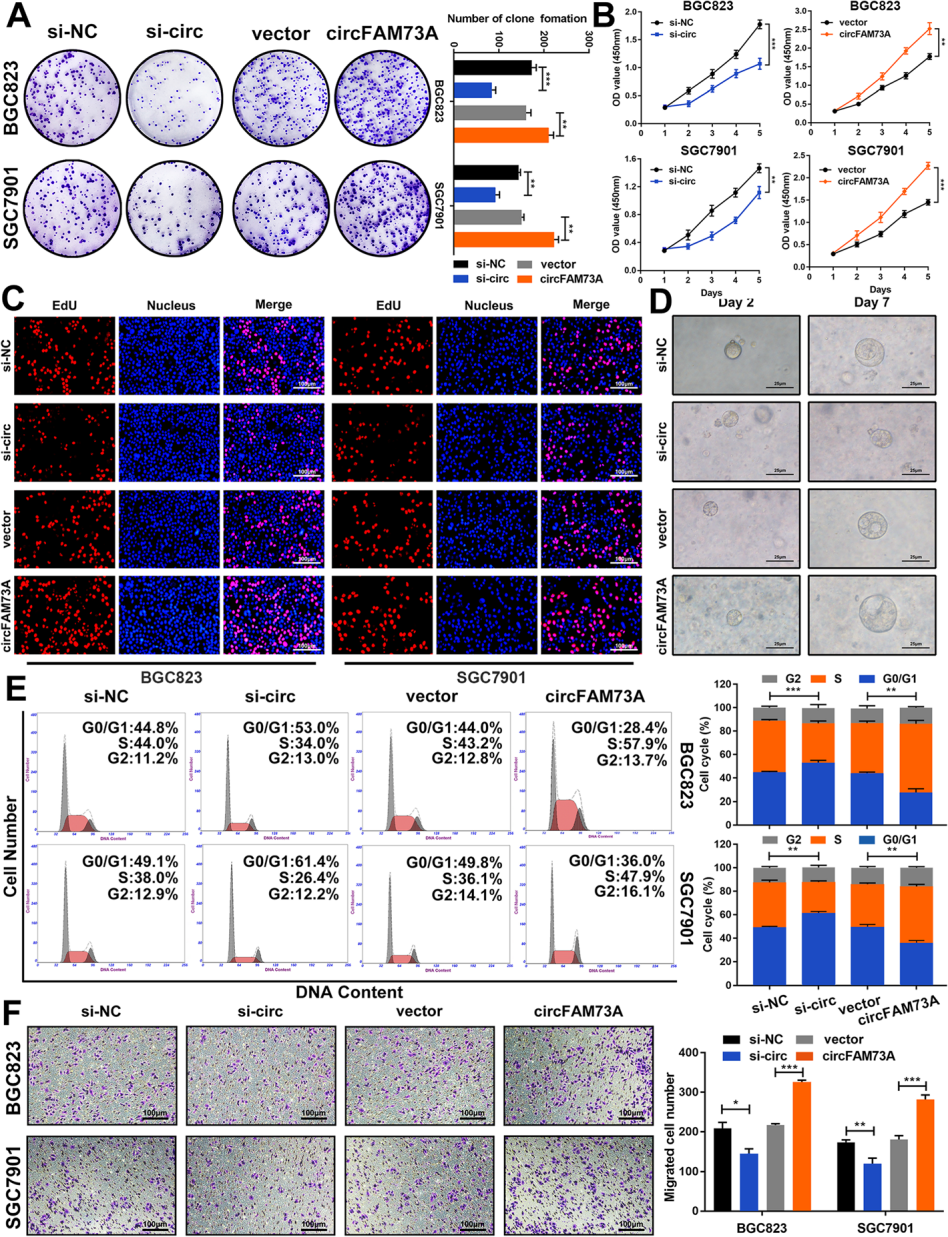
CircFAM73A promotes the proliferation and migration of GC in vitro*.* **a** Colony formation assay of BGC823 and SGC7901 transfected with control, circFAM73A siRNA, vector or circFAM73A plasmid. **b, c** CCK-8 and EdU assays were performed to evaluate cell proliferative effects of circFAM73A in BGC823 and SGC7901. Scale bar: 100 μm. **d** Effects of circFAM73A alteration on the growth of gastric organoids. Scale bar: 25 μm. **e** Effects of circFAM73A alteration on cell cycle distribution of BGC823 and SGC7901 detected by flow cytometry. **f** Effects of circFAM73A alteration on cell migration ability were tested by Transwell assay. Scale bar: 100 μm. Graph represents mean ± SD; *p < 0.05, **p < 0.01, ***p < 0.001

We first performed colony formation and CCK-8 assays to examine the effects of circFAM73A on cell proliferation. The results showed that transfection with siRNA dramatically suppressed the proliferation of BGC823 and SGC7901 cells. In contrast, exogenous expression of circFAM73A exerted the opposite effects (Fig. 2a and b). Next, a 5-ethynyl-2′-deoxyuridine (EdU) incorporation assay also demonstrated that circFAM73A increased the rate of EdU incorporation in cells (Fig. 2c and S2d). A 3D GC organoid model was then established to further test the proliferative ability. We found that silencing circFAM73A significantly decreased the diameter of organoids, and the opposite findings were found in the circFAM73A reconstitution groups (Fig. 2d and S2e). To determine whether circFAM73A interfered with the cell cycle, we then assessed the cell cycle distribution using flow cytometry. As shown in Fig. 2e, interference with circFAM73A expression distinctly increased the percentage of G0/G1 phase cells and diminished the percentage of S phase cells, while overexpressing circFAM73A showed the opposite trend. Western blot demonstrated that circFAM73A overexpressing elevated the expression of cyclin proteins related to G1/S transition, including cyclin D1, cyclin E1 and CDK2, while circFAM73A knockdown decreased these proteins (Fig. S2f and S2g). These observations indicated that interference with circFAM73A induced cell cycle arrest in the G0/G1 phase, thereby constraining the proliferation of GC cells. Moreover, cell migration was impeded by circFAM73A depletion and promoted by overexpressing circFAM73A in both BGC823 and SGC7901 cells based on Transwell assays (Fig. 2f).

For advanced or metastatic GC patients, chemotherapy based on cis-dichlorodiammine platinum (CDDP)/cisplatin is currently recommended as the first-line therapy. We therefore assessed the effects of circFAM73A on the chemosensitivity of GC cells. BGC823 and SGC7901 cells resistant to CDDP (referred to as BGC823CDDP and SGC7901CDDP cells) were established as described previously [24]. As shown in Fig. S3a, circFAM73A expression was considerably increased in the BGC823CDDP and SGC7901CDDP cells compared with the corresponding sensitive cells by qRT-PCR. To test whether circFAM73A modulated the sensitivity of GC cells to cisplatin treatment, we treated cells with various concentrations of CDDP. CircFAM73A overexpression enhanced cell viability and elevated the IC50 in the BGC823 and SGC7901 cells (Fig. S3b and S3c), whereas a reduction in circFAM73A in the BGC823CDDP and SGC7901CDDP cells showed the opposite effects (Fig. S3d and S3e). Our colony formation assays demonstrated that upregulation of circFAM73A enhanced the long-term viability of the CDDP-sensitive cells. In comparison, downregulation of circFAM73A displayed the opposite results in the CDDP-resistant cells (Fig. S3f). Moreover, the results of the flow cytometric assay showed that circFAM73A reduced the apoptosis rates of the GC cells treated with CDDP (Fig. S3g).

Collectively, these findings demonstrated that circFAM73A promotes GC cell proliferation and migration and facilitates cisplatin resistance in vitro.

### CircFAM73A enhances stem cell-like properties in GC cells

A growing number of studies suggest that the acquisition of cancer stem cell-like properties is crucial for the initiation and maintenance of the malignancy of GC [3, 4]. Given the facilitating role of circFAM73A in cell proliferation and cisplatin resistance, we asked whether circFAM73A enhances the stem cell-like properties of GC cells.

**Fig. 3:**
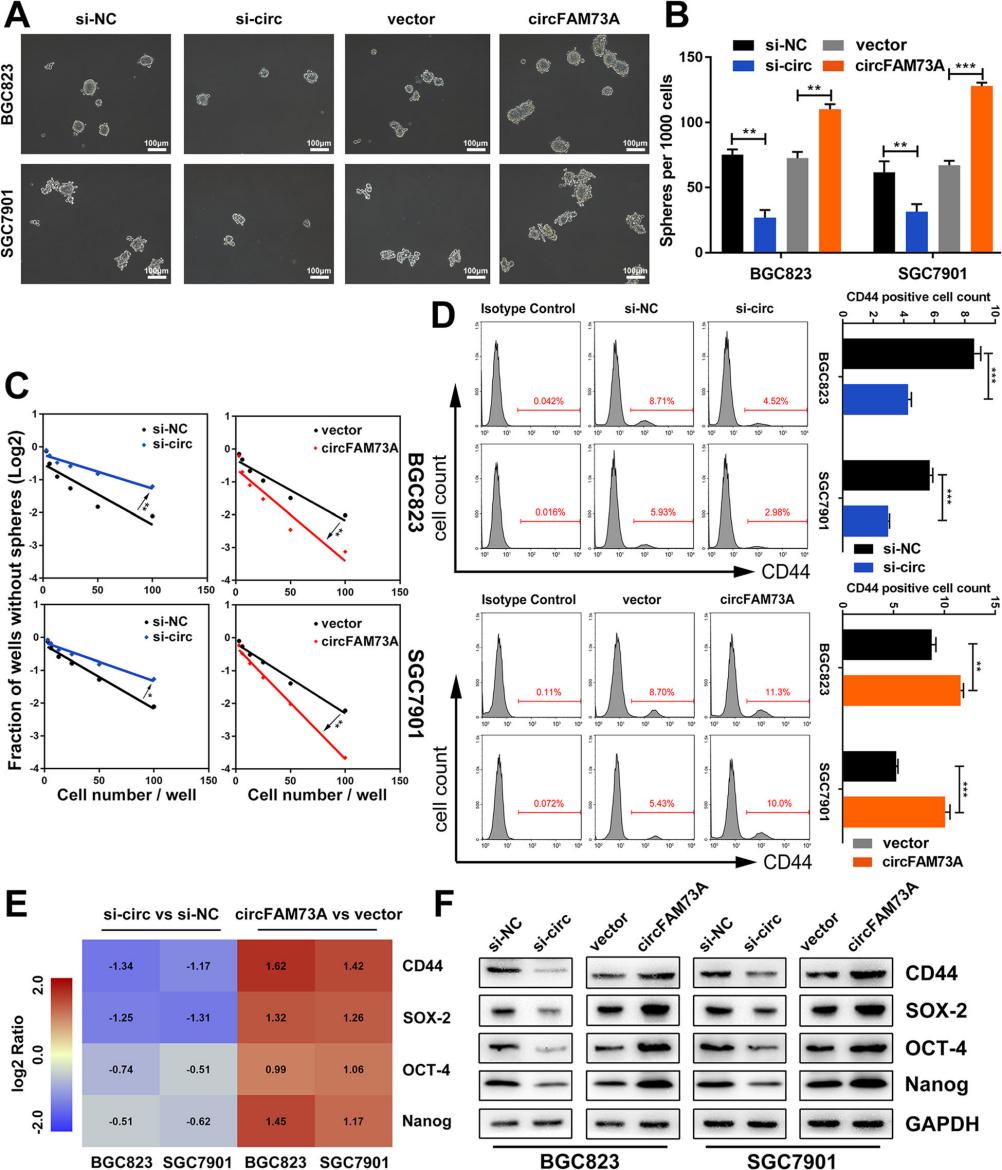
CircFAM73A enhances the stem cell-like property in GC cells. **a** Representative images of formatted spheres among indicated cells. Scale bar: 100 μm. **b** Sphere formation abilities were accessed by the number of spheres. **c** Effects of circFAM73A alteration on capacity of sphere formation were measured by extreme limiting dilution assay. **d** Representative flow cytometric histograms and quantification of the CD44 positive proportion among indicated cells. **e** Several stemness-related factors including CD44, SOX-2, OCT-4 and Nanog were measured by qRT-PCR. Pseudocolors represent the intensity scale of expression in circFAM73A vs. vector cells and si-circFAM73A vs. control cells calculated by log2 transformation. **f** Western blot of stemness-related factors including CD44, SOX-2, OCT-4, and Nanog among indicated cells. Graph represents mean ± SD; *p < 0.05, **p < 0.01, ***p < 0.001

We first explored the role of circFAM73A in GC cell self-renewal. To this end, we performed a sphere formation assay. As shown in Fig. 3a and b, overexpression of circFAM73A promoted the generation and cell content of tumor spheres of the BGC823 and SGC7901 cells cultured in suspension. Conversely, the circFAM73A-silenced cells formed fewer spheres with lower cell content. The circFAM73A-mediated induction of the self-renewal ability was also confirmed using a limiting dilution assay (Fig. 3c).

In addition, augmentation of circFAM73A increased, while downregulation of circFAM73A reduced, the proportion of CD44 (GC stem cell-like marker)-positive BGC823 and SGC7901 cells (Fig. 3d). qRT-PCR and Western blot analysis showed that circFAM73A significantly increased the expression of CD44 and stemness-associated transcription factors, including SOX-2, OCT-4 and Nanog. The opposite results were found in the circFAM73A-depressed cells (Fig. 3e, f, and S3h).

Together, these findings indicated that circFAM73A exhibits a positive effect on CSC-like properties in GC cells.

### CircFAM73A acts as a sponge of miR-490-3p, and HMGA2 is the direct downstream target of miR-490-3p

CircRNAs function primarily as miRNA sponges by sequestering specific miRNAs, resulting in changes in specific gene expression. We therefore investigated the potential miRNAs associated with circFAM73A.

**Fig. 4:**
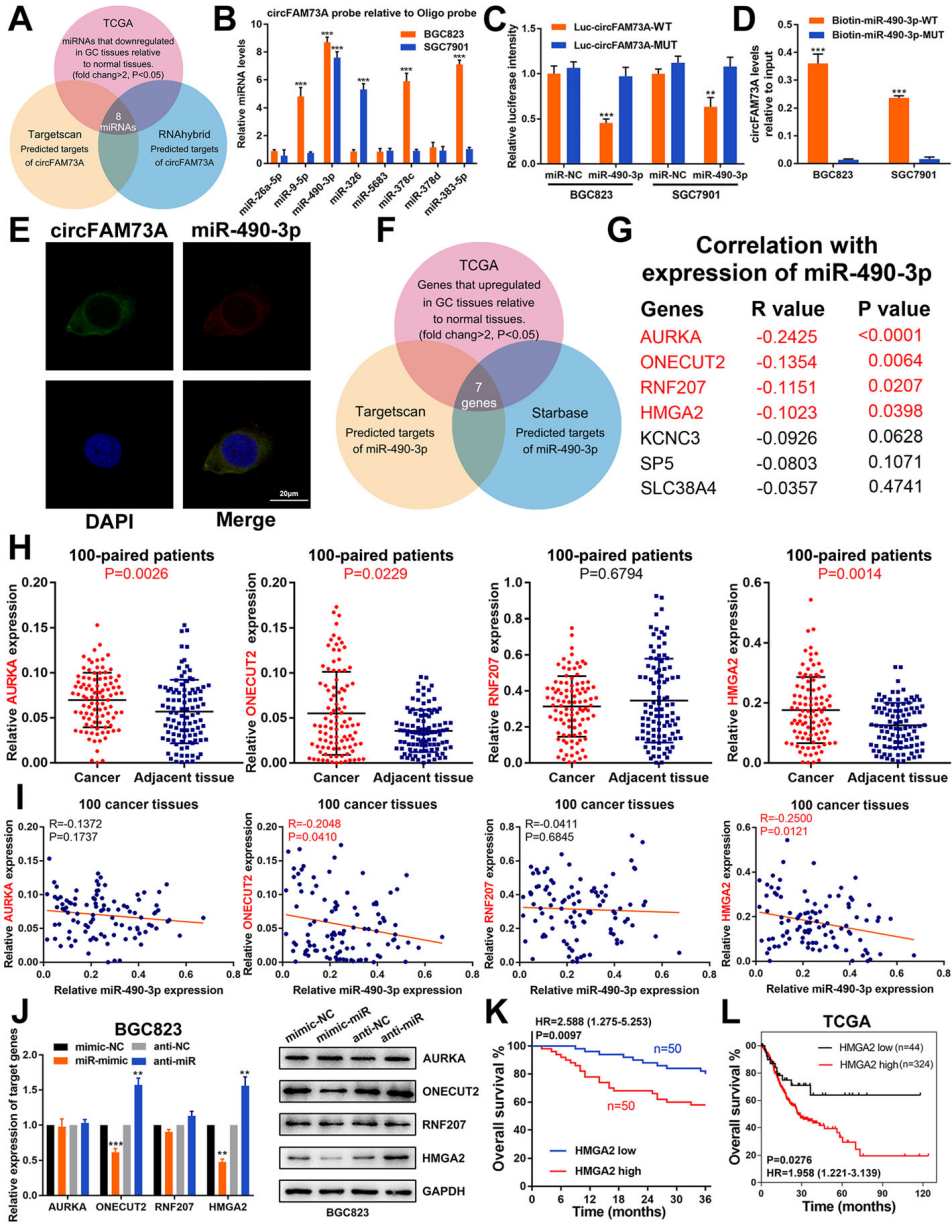
CircFAM73A acts as a sponge of miR-490-3p, HMGA2 is the direct downstream target of miR-490-3p. **a** Venn diagram showing the overlap of target miRNAs of circFAM73A predicted by TargetScan and RNAhybrid and downregulated miRNAs in GC from TCGA. **b** The expression levels of 8 miRNAs candidate miRNAs were quantified by qRT-PCR after pull-down with biotin-labeled circFAM73A probe in BGC823 and SGC7901 cells. **c** Luciferase intensity in BGC823 and SGC7901 cells co-transfected with luciferase reporter containing with wild-type or mutated circFAM73A-miR-490-3p binding sequences and the mimics of miR-490-3p or control. **d** The expression levels of circFAM73A were tested by qRT-PCR after pull-down with biotin-labeled wild-type or mutant miR-490-3p in circFAM73A overexpressed BGC823 and SGC7901 cells. **e** Fluorescence in situ hybridization assay revealed the co-location between circFAM73A and miR-490-3p in cytoplasm. Scare bar = 20 μm. **f** Venn diagram detailing the exploration of miR-490-3p downstream targets. **g** The correlation between miR-490-3p and the 7 potential targets gene in TCGA database. **h** The mRNA expression of AURKA, ONECUT2, RNF207, and HMGA2 in 100 paired GC and adjacent tissues were determined by qRT-PCR. **i** Correlation between miR-490-3p and AURKA, ONECUT2, RNF207, and HMGA2 according to our GC samples. **j** The expression of AURKA, ONECUT2, RNF207, and HMGA2 after miR-490-3p alternation in BGC823 cells measured by qRT-PCR and Western Blot. **k** Overall survival analysis based on HMGA2 expression in 100 GC patients. The median HMGA2 expression is used as a cutoff. **l** Overall survival analysis based on HMGA2 expression in TCGA GC patients. The optimal cut-off was calculated by X-tile software. Graph represents mean ± SD; *p < 0.05, **p < 0.01, ***p < 0.001

Given the elevated expression and the promotive effects of circFAM73A in GC, we screened the predicted miRNA targets obtained from TargetScan and RNAhybrid together with miRNAs that were significantly downregulated in GC samples according to TCGA database (fold change > 2, *p* < 0.05) (Fig. 4a). We identified 8 candidate miRNAs that matched these criteria (Fig. S4a). A biotin-labeled circFAM73A probe was then designed to examine the potential miRNAs that interacted with circFAM73A. The probe efficiency was verified in GC cells, while circFAM73A overexpression further enhanced the pulldown efficiency (Fig. S4b). As shown in Fig. 4b, qRT-PCR revealed that miR-490-3p was the only miRNA that was pulled down by the circFAM73A probe in both BGC823 and SGC7901 cells. To further verify the direct binding of circFAM73A and miR-490-3p, we performed luciferase reporter assays, which demonstrated that overexpression of miR-490-3p substantially decreased the luciferase activity of the reporter containing the wild-type circFAM73A sequence but had no effects on the reporter containing circFAM73A with the mutant miR-490-3p-binding site in BGC823 and SGC7901 cells (Fig. 4c). Furthermore, compared with the mutant biotin-labeled miR-490-3p, wild-type miR-490-3p captured more circFAM73A in GC cells with circFAM73A overexpression (Fig. 4d), indicating the binding between circFAM73A and miR-490-3p. FISH assays showed colocalization between circFAM73A and miR-490-3p in the cytoplasm (Fig. 4e). A reduction in miR-490-3p in GC tissues was also found in our samples (Fig. S4c). These results suggested that circFAM73A exerts its effects by sponging miR-490-3p.

To identify miR-490-3p target genes, we screened starBase and TargetScan for the predicted targets of miR-490-3p and combined with the genes that are significantly upregulated in TCGA GC database (fold change > 2, *p* < 0.05) (Fig. 4f). Using this strategy, we identified 7 genes. We then analyzed the correlations between miR-490-3p expression and these 7 candidate targets in TCGA GC database. Four genes (AURKA, ONECUT2, RNF207, and HMGA2) showed clear negative correlations with miR-490-3p (Fig. 4g and S4d) and were therefore chosen for further experiments.

qRT-PCR verified the upregulation of AURKA, ONECUT2, and HMGA2 in our 100 paired GC tissues compared with adjacent tissues, whereas no significant changes in RNF207 expression were observed (Fig. 4h). Moreover, the ONECUT2 and HMGA2 expression levels exhibited a clear negative correlation with miR-490-3p, while we found no such correlation for AURKA and RNF207 (Fig. 4i). As shown in Fig. 4j and S4e-S4g, overexpression of miR-490-3p reduced the expression of ONECUT2 and HMGA2; in comparison, inhibition of miR-490-3p increased the expression of ONECUT2 and HMGA2. However, the expression of AURKA and RNF207 showed no change after miR-490-3p overexpression or inhibition. Next, siRNAs specific to these four genes were transfected into GC cells. Only the interference sequence of AURKA and HMGA2 suppressed the viability of BGC823 and SGC7901 cells, as determined by CCK-8 assays (Fig. S4h).

On the basis of these results, we chose HMGA2, a potential tumor promoter and target of miR-490-3p in GC, for further studies. The flow chart of bioinformatic analysis to identify miR-490-3p target genes was shown in Fig. S4i.

Luciferase reporter assays confirmed that HMGA2 was a direct downstream target of miR-490-3p (Fig. S4j). Moreover, rescue assays were conducted which further confirming the regulation of miR-490-3p on HMGA2 (Fig. S5). Importantly, Kaplan–Meier survival analysis of our 100 GC patients and TCGA database showed that higher HMGA2 expression correlated with poor overall survival of GC patients (Fig. 4k and l).

### CircFAM73A regulates HMGA2 expression by miR-490-3p

**Fig. 5:**
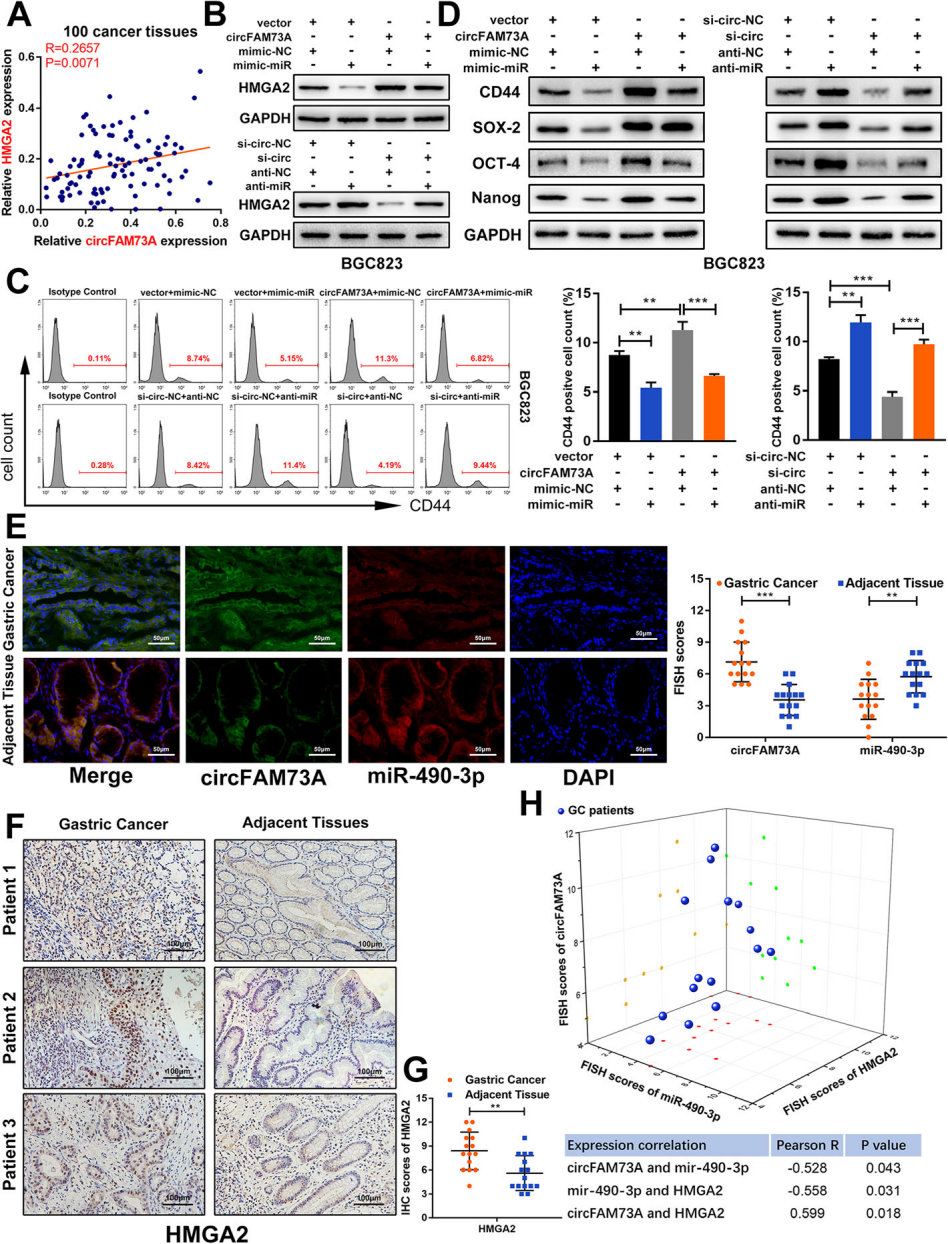
CircFAM73A regulates HMGA2 expression by miR-490-3p. **a** Correlation between circFAM73A and HMGA2 according to our GC samples. **b** Western blot of HMGA2 was detected in BGC823 transfected with indicated vectors. **c** Representative flow cytometric histograms and quantification of the CD44 positive proportion in BGC823 transfected with indicated vectors. **d** Western blot of stemness-related factors including CD44, SOX-2, OCT-4, and Nanog in BGC823 transfected with indicated vectors. **e** FISH assay of circFAM73A and miR-490-3p in 15 paired GC and adjacent tissues from patients. FISH scores were further assessed. Nuclei were stained with DAPI. Scale bar: 50 μm. **f** IHC staining of HMGA2 in in 15 paired GC and adjacent tissues. Scale bar: 100 μm. **g** The IHC scores of HMGA2 were further quantified. **h** Three-dimensional scatter plot of circFAM73A, miR-490-3p and HMGA2 15 paired GC and adjacent tissues. Graph represents mean ± SD; *p < 0.05, **p < 0.01, ***p < 0.001

High mobility group A2 (HMGA2) is a stem cell factor primarily expressed during embryogenesis, with low abundance in adult human tissues [25]. HMGA2 is aberrantly expressed in several types of cancer, with high levels of HMGA2 associated with a highly malignant phenotype, as it is related to increased tumor proliferation, invasiveness, and stemness and reduced survival [26–28]. As shown in Fig. 5a, a positive correlation between the mRNA levels of circFAM73A and HMGA2 was detected in GC tissues, indicating the potential regulation of HMGA2 by circFAM73A in GC.

To assess whether circFAM73A regulates HMGA2 expression by miR-490-3p, we reduced miR-490-3p expression in the circFAM73A-repressing cells and elevated miR-490-3p in the circFAM73A-overexpressing cells. Our Western blot and qRT-PCR data showed that circFAM73A increased the expression of HMGA2, while the miR-490-3p mimic attenuated this effect. In contrast, the reduction in HMGA2 caused by circFAM73A inhibition was also reversed by miR-490-3p knockdown in both BGC823 and SGC7901 cells (Fig. 5b, Fig. S6a-S6c). Moreover, flow cytometry demonstrated that exogenous circFAM73A expression increased the proportion of CD44-positive cells, and this effect was reversed by the miR-490-3p mimic. In comparison, circFAM73A suppression led to a decline in CD44-positive cells, which was also reversed by miR-490-3p inhibition (Fig. 5c and S6d). The same results were also acquired when we examined the effects of circFAM73A on other stemness-associated transcription factors (SOX-2, OCT-4, and Nanog) by Western blots (Fig. 5d and S6e-S6g).

In addition, FISH assays showed colocalization between circFAM73A and miR-490-3p in GC tissues. FISH scores confirmed that the expression of circFAM73A was higher in the GC tissues than in the matched noncancerous tissues, whereas miR-490-3p expression showed the opposite change (Fig. 5e). Similarly, IHC staining also demonstrated the increase in HMGA2 protein levels in GC tissues (Fig. 5f and g). Importantly, we found that circFAM73A, miR-490-3p, and HMGA2 were well correlated in these GC tissues (Fig. 5h).

These observations indicated that circFAM73A regulates HMGA2 expression and stemness-related pathways by sponging miR-490-3p.

### CircFAM73A promotes stem cell-like properties and cell malignancy in GC cells by upregulating HMGA2 expression

Based on the results above, we hypothesized that circFAM73A improves stem cell-like properties and cell malignancy in GC by sponging miR-490-3p, thus upregulating HMGA2 expression. To test this hypothesis, we either repressed or reconstituted HMGA2 expression in the circFAM73A-overexpressing or circFAM73A-silenced cells, respectively.

**Fig. 6:**
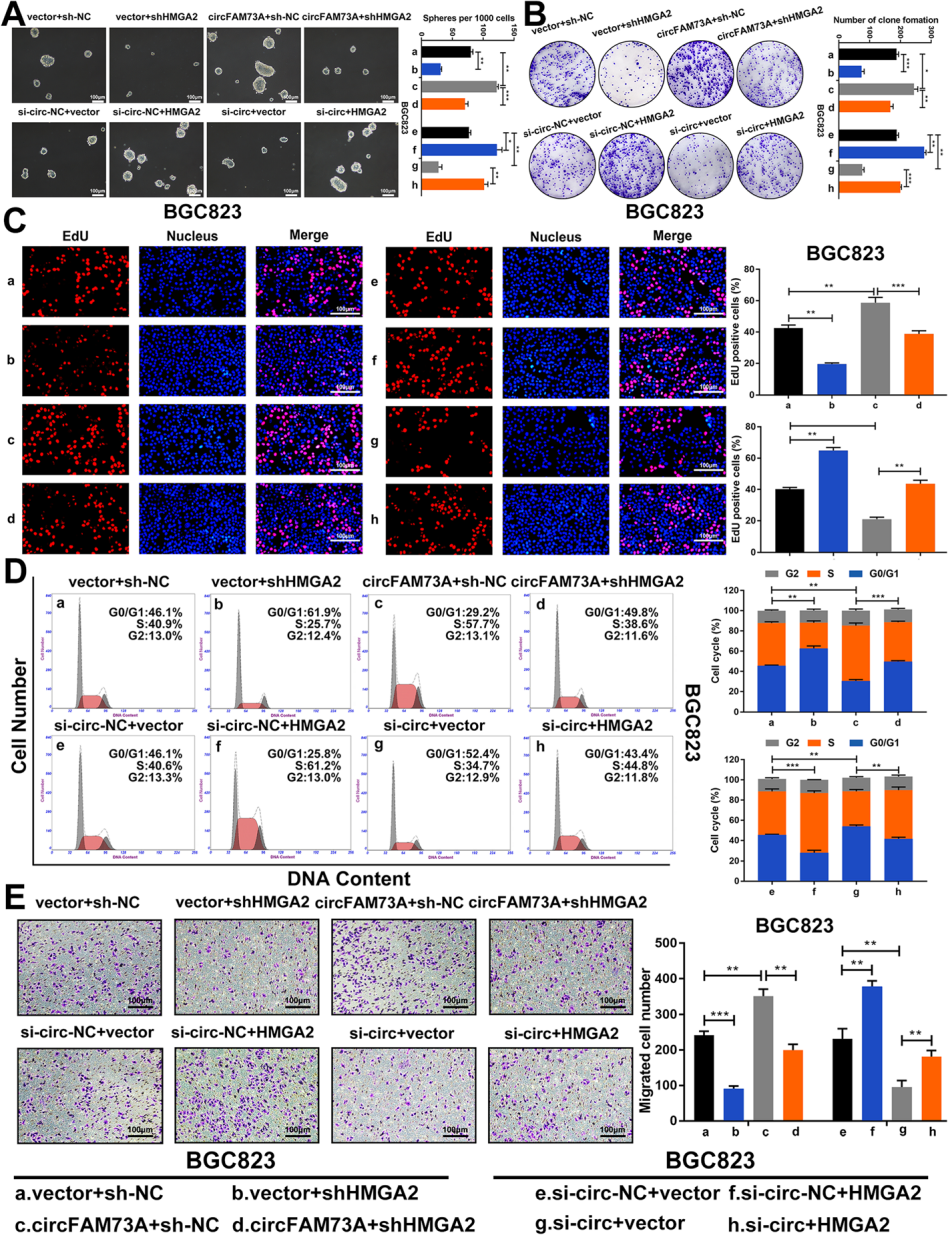
CircFAM73A promotes stem cell-like property and cell malignancy in GC cells by upregulating HMGA2 expression. **a** Representative images and quantification of formatted spheres among indicated cells. Scale bar: 100 μm. **b** Representative images and quantification of clone formation among indicated cells. **c** Representative images of EdU staining and quantification of EdU positive cells among indicated cells. Scale bar: 100 μm. **d** Representative images of cell cycle distribution among indicated cells detected by flow cytometry. **e** Representative images and quantification of migrated cells among indicated cells tested by Transwell assay. Scale bar: 100 μm. Graph represents mean ± SD; *p < 0.05, **p < 0.01, ***p < 0.001

Using a sphere formation assay, we found that the circFAM73A-mediated induction of cell self-renewal was counteracted by HMGA2 downregulation. HMGA2 reconstitution reversed the inhibitory effect of circFAM73A repression (Fig. 6a and S7a). Similarly, HMGA2 downregulation also impaired the cell proliferation and migration caused by exogenous circFAM73A expression, and ectopic HMGA2 expression reversed the inhibitory effects of circFAM73A suppression based on the results of further experiments, including colony formation assays (Fig. 6b and S7b), EdU assays (Fig. 6c and S7c), flow cytometry (Fig. 6d and S7d) and Transwell assays (Fig. 6e and S7e).

Together, these results confirmed that circFAM73A promotes cancer stem cell-like properties and cell malignancy in GC cells by upregulating HMGA2 expression.

### CircFAM73A regulates HMGA2 expression levels to promote GC growth and metastasis in vivo

**Fig. 7:**
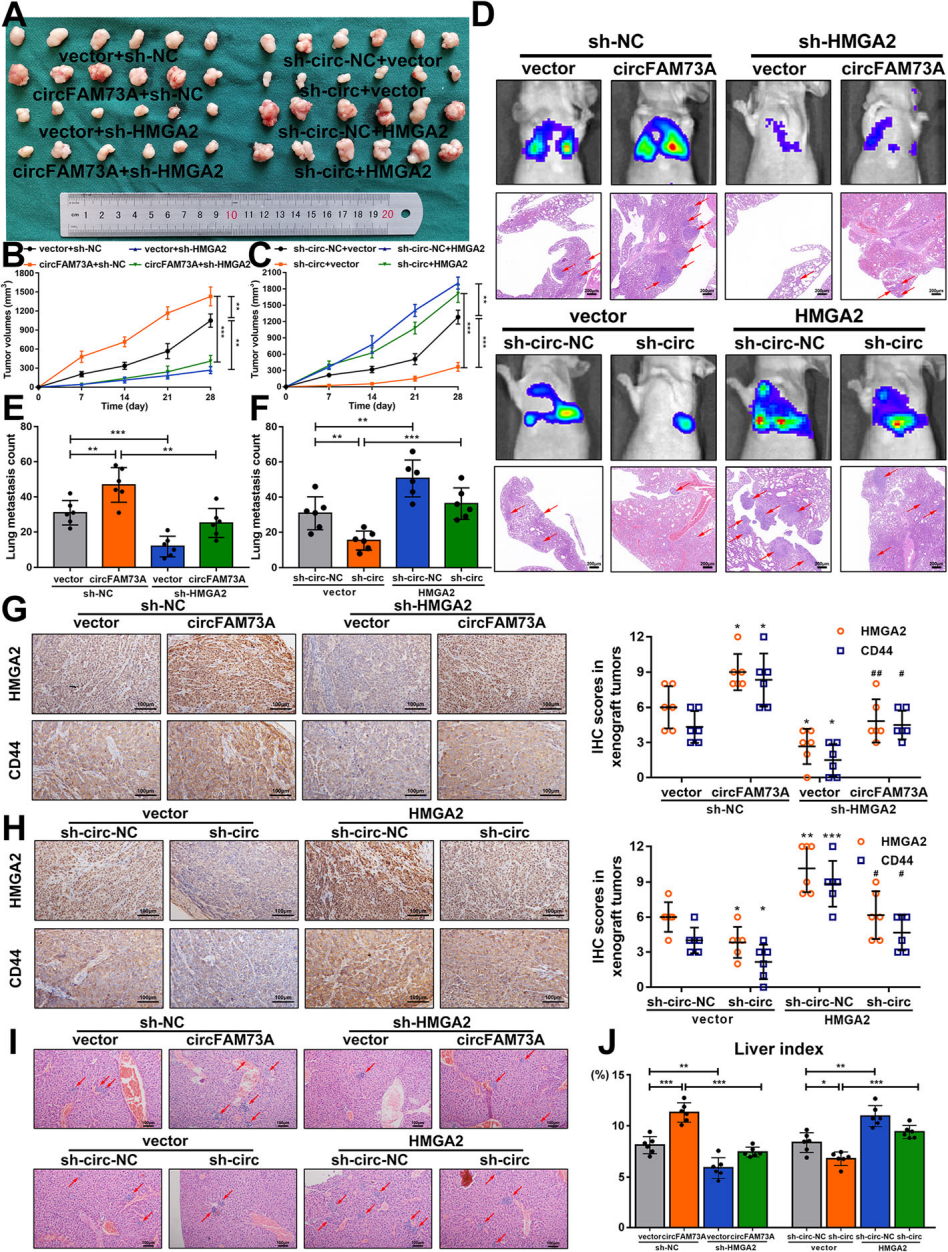
CircFAM73A regulates HMGA2 expression level to promote GC growth and metastasis in vivo*.* **a** The images of xenograft tumors of sacrificed mice subcutaneous injected with indicated cells 4 weeks after injections. **b, c** Growth curves of xenograft tumors. Tumor volume was calculated by 1/2 (length × width2). **d** Representative images of bioluminescent images of mice by IVIS Imaging system and HE staining of lung tissues. Scale bar: 200 μm. **e** Quantification of metastatic foci in mice lungs injected with indicated cells. **g, h** Immunohistochemistry staining and IHC scores of HMGA2 and CD44 in respective xenograft tumor tissues. * vs the group of first column, ^#^ vs the group of second column. **i** Representative images of HE staining of liver tissues from mice with portal veins injection. Scale bar: 100 μm. **j** Liver indexes (liver weight/ body weight) of each group were calculated. Graph represents mean ± SD; *p < 0.05, **p < 0.01, ***p < 0.001, ^#^*p* < 0.05, ^# #^*p* < 0.01, ^# # #^*p* < 0.001

To delineate the roles of circFAM73A and HMGA2 in vivo, we first generated xenograft tumors in nude mice. Xenograft tumors generated from the circFAM73A-overexpressing BGC823 cells showed considerably faster growth, whereas the circFAM73A-suppressed xenografts were smaller in volume than those formed from the control cells. Moreover, repressing HMGA2 expression reversed the positive effect of circFAM73A on xenograft tumor formation, while exogenous expression of HMGA2 attenuated the effects caused by circFAM73A disruption (Fig. 7a-c, S8a, and S8b). Our qRT-PCR results showed that circFAM73A increased the mRNA levels of HMGA2 and CD44 in xenograft tumor samples, an effect that was reversed by HMGA2 depletion (Fig. S8c). In contrast, exogenous HMGA2 expression reversed the reduction caused by cirFAM73A suppression (Fig. S8d). Immunohistochemistry staining of HMGA2 and CD44 in xenograft tumors demonstrated the same effects (Fig. 7g and h).

To examine the metastatic abilities of circFAM73A and HMGA2, we established two in vivo models. In the lung metastasis model, the tumor cells were injected into the caudal veins of mice, and metastasis was monitored by an IVIS imaging system. After 6 weeks, the mice were euthanized. Lung tissues were obtained for HE staining (Fig. 7d), and lung metastatic foci were quantified (Fig. 7e and f). In the liver metastasis model, tumor cells were injected into the portal veins of mice. Livers were harvested after 6 weeks and stained with HE (Fig. 7i). The number of nodes was counted (Fig. S8e and S8f), and the liver indexes (liver weight/body weight) were calculated (Fig. 7j). These two models clearly showed that circFMA73A enhanced the metastasis of GC in vivo*,* which was also mediated by HMGA2.

Together, these data confirmed that circFAM73A promotes both GC growth and metastasis in vivo by regulating HMGA2 expression levels.

### HMGA2 enhances the transcriptional activation of FAM73A by E2F1 and elevates the efficiency of circFAM73A circularization by HNRNPL, which in turn elevates circFAM73A expression

HMGA2 functions as an architectural transcription modulator and facilitates the function of several transcription factors, including E2F1. Previous studies revealed that the interaction between HMGA2 and pRB enhances the activation of E2F1 in pituitary adenomas [26]. We then investigated whether HMGA2 facilitates the function of E2F1 in GC.

**Fig. 8:**
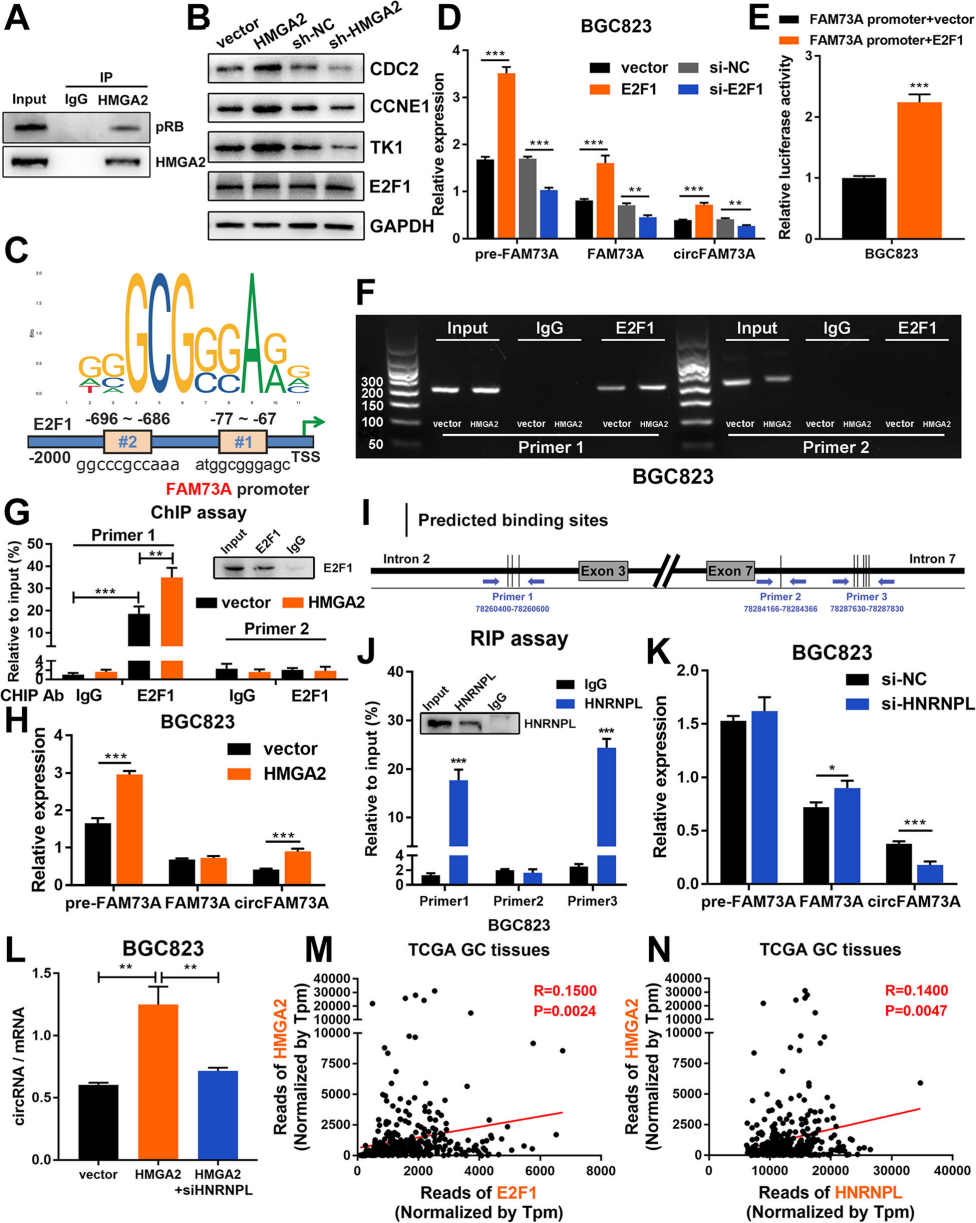
HMGA2 elevates circFAM73A expression reciprocally by E2F1 and HNRNPL. **a** Co-immunoprecipitation was performed using control IgG or HMGA2 antibody. Immunoprecipitated proteins were detected by pRB and HMGA2 antibodies. **b** Western blot of E2F1 and classical E2F1 responsive effectors (CCNE1, TK1, and CDC2) in BGC823. **c** Putative binding sites of E2F1 in FAM73A promoter region predicted by JASPAR. **d** Relative expression of pre-FAM73A, FAM73A mRNA, and circFAM73A with E2F1 alteration were measured by qRT-PCR in BGC823. **e** Luciferase reporter assay analysis of FAM73A promoter luciferase reporters in BGC823 cells transfected with E2F1 or control. **f** RT-PCR was performed in BGC823 cells after chromatin immunoprecipitation by E2F1 antibody or control IgG and by two pairs of primers to validate the E2F1 binding sites in FAM73A promoter region. **g** qRT-PCR analysis of chromatin immunoprecipitation assay in E. **h** Relative expression of pre-FAM73A, FAM73A mRNA, and circFAM73A in BGC823 with HMGA2 reconstitution were measured by qRT-PCR. **i** Schematic diagram demonstrated three designed specific primers covering different binding site sequences in intron-2 and intron-7. **j** Relative enrichment of amplification sequence by three indicated primes after RNA binding protein immunoprecipitation assay by HNRNPL antibody or control IgG in BGC823. **k** Relative expression of pre-FAM73A, FAM73A mRNA, and circFAM73A in BGC823 with HNRNPL suppression were measured by qRT-PCR. **l** The ratio of circFAM73A expression to FAM73A mRNA expression in BGC823. **m** Correlation between E2F1 and HMGA2 according to TCGA statistics. **n** Correlation between HNRNPL and HMGA2 according to TCGA statistics. Graph represents mean ± SD; *p < 0.05, **p < 0.01, ***p < 0.001

First, immunoprecipitation assays confirmed the interactions between HMGA2 and pRB in GC cells (Fig. 8a). We then examined the effects of HMGA2 on E2F1 activity by measuring the expression of several classic E2F1-responsive effectors (CDC2, CCNE1, and TK1). Western blots and qRT-PCR demonstrated that both the protein and mRNA levels of these effectors were increased by ectopic expression of HMGA2 and decreased by HMGA2 repression (Fig. 8b, S9a-S9c). However, no change in E2F1 expression was detected upon HMGA2 alteration, suggesting that HMGA2 might simply increase the activity of E2F1 rather than its expression in GC.

Intriguingly, two putative binding sites of E2F1 were found in the FAM73A promoter region using JASPAR (#1 ATGGCGGGAGC, − 77 to − 67, #2 GGCCCGCCAAA, − 696 to − 686) (Fig. 8c). We therefore investigated whether E2F1 promotes the transcription of FAM73A and results in an increase in FAM73A mRNA or circFAM73A. qRT-PCR by specific primers (Fig. S9d) showed that the pre-FAM73A, FAM73A, and circFAM73A levels were increased following E2F1 overexpression but suppressed upon E2F1 depletion (Fig. 8d and S9e). To examine the binding of E2F1 to the FAM73A promoter, we performed a luciferase reporter assay. As shown in Fig. 8e and S9f, E2F1, rather than the vector plasmid, elevated the luminescence of the luciferase reporter containing the FAM73A promoter region. ChIP-PCR analysis using a specific antibody against E2F1 confirmed the occupancy of E2F1 on binding site #1 in the FAM73A promoter region. Moreover, this effect could be promoted by ectopic HMGA2 expression (Fig. 8f, g, S9g, and S9h), indicating that HMGA2 enhanced the transcription of FAM73A by E2F1. As expected, HMGA2 increased both pre-FAM73A and circFAM73A production. However, elevated pre-FAM73A caused by HMGA2 induction only resulted in an increase in circFAM73A but not linear FAM73A mRNA (Fig. 8h and S9i).

The uncorrelated levels of circular RNAs and linear mRNAs indicated that HMGA2 might also affect the post-transcriptional processing and increase the backsplicing efficiency in pre-FAM73A, making circular RNAs the preferred gene output over linear RNAs. Mounting evidence suggests that splicing factors contribute to circRNA biogenesis post-transcriptionally by targeting specific sequences within flanking introns and drawing backsplicing exon ends into close proximity [20, 29, 30]. We then utilized the MEME Suite [31] to analyze the known RBP binding motifs flanking intron-2 and intron-7 of circFAM73A (*p* < 0.001, q < 0.5). Several RBP motifs were found, and among them, HNRNPL was predicted to harbor binding sites on both intron-2 and intron-7 (Fig. S9j) and was previously confirmed to promote circRNA circularization [32].

To identify the direct HNRNPL binding sites in the flanking intron of circFAM73A, we performed RNA-immunoprecipitation (RIP) assays using qRT-PCR to quantify HNRNPL occupancy within the introns adjacent to the circFAM73A-forming exons. Three pairs of primers were designed according to the potential binding sequences. Primer 1 contains the three predicted HNRNPL binding sites in intron-2. Primer 2 targets the first predicted binding sites in intron-7, and the 2 to 6 predicted binding sites in intron-7 cover the amplification sequence of Primer 3 (Fig. 8i). We found that HNRNPL bound to the Primer 1 region in intron-2 and the Primer 3 region in intron-7 but not the Primer 2 region (Fig. 8j and S9k). Furthermore, knockdown of HNRNPL resulted in a 132#?>significant reduction in circFAM73A but not pre-FAM73A. In addition, we observed a small but significant increase in FAM73A mRNA (Fig. 8k and S9l). Together, these findings demonstrated that HNRNPL binds to the flanking intron region and elevates circFAM73A formation.

We then investigated whether HMGA2 promotes the circularization of circFAM73A by HNRNPL. The ratio of circRNA expression to mRNA expression was calculated to reflect the circularization efficiency. As shown in Fig. 8l and S9m, exogenous HMGA2 expression resulted in increased circularization efficiencies, which were reduced when HNRNPL was additionally suppressed, suggesting that HMGA2 enhances the efficiency of circFAM73A circularization by HNRNPL.

In addition, we found that both E2F1 and HNRNPL correlated well with HMGA2 in TCGA database (Fig. 8m and n), which indicated that E2F1 and HNRNPL regulate circFAM73A and its downstream target HMGA2.

Collectively, these results demonstrated that HMGA2 plays a dichotomous role in regulating circFAM73A expression, i.e., HMGA2 facilitates the transcriptional activation of FAM73A by E2F1 and elevates the efficiency of circFAM73A circularization by HNRNPL.

### CircFAM73A directly interacts with HNRNPK and facilitates β-catenin stabilization

**Fig. 9:**
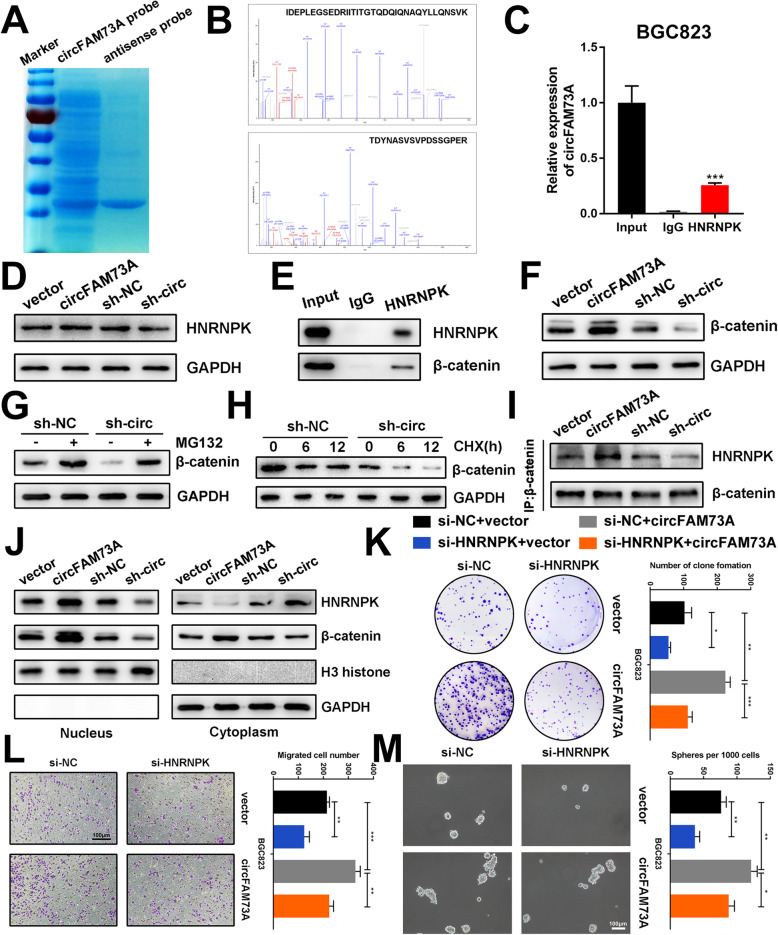
CircFAM73A interacts with HNRNPK and facilitates β-catenin stabilization. **a** Coomassie blue staining of circFAM73A pulldown. **b** Spectra of HNRNPK identified by mass spectrometry. **c** Relative abundance of circFAM73A detected by qRT-PCR after RIP using HNRNPK antibody in BGC823. **d** Expression of HNRNPK protein of BGC823 after circFAM73A overexpression or knocking-down measured by Western Blot. **e** Co-IP analysis using HNRNPK protein revealing the endogenous interaction between HNRNPK and β-catenin in BGC823. **f** Expression of β-catenin protein in BGC823 after circFAM73A overexpression or knocking-down measured by Western Blot. **g** Expression of β-catenin protein in BGC823 transfected with control shRNA or sh-circFAM73A and treated with MG132 (10 μmol/L, 10 h) or untreated measured by Western Blot. **h** Expression of β-catenin protein in BGC823 transfected with control shRNA or sh-circFAM73A and treated with cycloheximide (CHX, 50 μg/mL) for different time measured by Western Blot. **i** Co-IP and Western blot showing the interaction between HNRNPK and β-catenin after circFAM73A overexpression or knocking-down in BGC823. **j** The nuclear and cytoplasmic expression of HNRNPK and β-catenin measured by Western Blot after circFAM73A overexpression or knocking-down in BGC823. **k-m** Representative images of and quantification of clone formation (**k**) migrated cells (**l**) and formatted spheres (**m**) in BGC823 transfected with vector or circFAM73A plasmid and co-transfected with control siRNA or si-HNRNPK. Graph represents mean ± SD; *p < 0.05, **p < 0.01, ***p < 0.001

To further explore the mechanism of circFAM73A, we conducted an RNA pulldown assay using a specific biotin-labeled circFAM73A probe. Coomassie blue staining showed several bands of proteins compared with those of the antisense probe (Fig. 9a), while mass spectrometry analysis identified HNRNPK (Fig. 9b), one of the major pre-mRNA-binding proteins. The RIP assay confirmed the direct interaction between circFAM73A and HNRNPK (Fig. 9c and S10a). However, the expression of HNRNPK showed no obvious change upon circFAM73A overexpression or knockdown by Western blot analysis (Fig. 9d and S10b) of BGC823 and SGC7901 cells, indicating that circFAM73A might regulate the activity rather than the expression of HNRNPK.

It has been reported that several lncRNAs interact with HNRNPK and facilitate the HNRNPK-mediated stability of β-catenin. Co-IP analysis was performed and indicated the endogenous interaction between HNRNPK and β-catenin in GC cells (Fig. 9e and S10c). We therefore investigated whether circFAM73A regulates the expression of β-catenin by HNRNPK. Western blot analysis demonstrated that ectopic expression or knockdown of circFAM73A increased or decreased β-catenin levels (Fig. 9f and S10d). Moreover, the reduction in β-catenin caused by circFAM73A repression was abolished by MG132 treatment (Fig. 9g and S10e). In addition, after treatment with cycloheximide, Western blot assays showed that circFAM73A knockdown shortened the half-life of β-catenin (Fig. 9h and S10f), indicating that circFAM73A stabilizes β-catenin by reducing the degradation of the β-catenin protein. We then investigated the roles of circFAM73A in HNRNPK-mediated β-catenin stability and found that ectopic expression or knockdown of circFAM73A promoted and weakened the interaction between HNRNPK and β-catenin in GC cells (Fig. 9i and S10g), respectively. Moreover, overexpression or knockdown of circFAM73A resulted in an increase or decrease in nuclear translocation and β-catenin (Fig. 9j and S10h).

Furthermore, functional experiments showed that the promoting effects of circFMA73A were reversed upon HNRNPK interference (Fig. 9k-m and S10i-S9k), identifying HNRNPK as the downstream target of circFAM73A.

Taken together, our results indicated that circFAM73A directly interacts with HNRNPK and facilitates the stabilization of β-catenin.

### Discussion

Despite the clinical application and continuous progress of systemic treatments in gastric cancer, this disease remains a high-mortality malignancy [1]. Cancer stem cells (CSCs) represent the composition that holds the capacities of self-renewal and differentiation in cancer cells [2], and to some extent, the proposal of a CSC model explains the high postoperative relapse and the resistance of cancer under current systemic treatments [33]. Therefore, a deep understanding of the biology and regulatory mechanism of CSCs may contribute to the development of new classes of therapeutics in cancer. However, studies concentrated on the effects of circRNAs on cancer stem cell-like properties are still comparatively insufficient.

Here, by screening GEO datasets and verifying them in our samples, we identified a circRNA originating from exons 3, 4, 5, 6, and 7 of FAM73A (circFAM73A), whose expression is relatively upregulated in GC, and then focused our investigation on the role and potential mechanism of circFAM73A in GC malignancy. Further Cox multivariate survival analysis identified high circFAM73A expression as an independent prognostic factor for poor survival of GC patients, indicating its tumor-promoting effects. Further functional experiments demonstrated that circFAM73A reinforced the stem cell-like properties in GC, thus facilitating cell proliferation, migration, and cisplatin resistance.

The specific mechanisms of circRNA function in cancer progression have not been fully elucidated [34]. Most studies of circRNAs regard them as competing endogenous RNAs. This hypothesis suggests that circRNAs could serve as decoys of available miRNAs, functionally releasing the downstream targets of that set of miRNAs [35]. In the current study, we screened the predicted miRNAs obtained from TargetScan and RNAhybrid and combined them with the downregulated miRNAs in TCGA. Eight candidate miRNAs were selected, and miR-490-3p was further confirmed to be capable of binding to circFAM73A by subsequent experiments. Next, for miR-490-3p target genes, we screened TCGA for upregulated genes together with predicted targets from starBase and TargetScan. Following bioinformatic analysis and experimental verification, HMGA2 was confirmed to be downstream of circFAM73A/miR-490-3p.

HMG (high mobility group) proteins were initially discovered in embryonic stem cells as the most abundant small, chromatin-associated DNA-binding protein [36]. However, HMGA2 expression becomes more restricted during fetal development, partly due to increasing let-7b expression [37] and is particularly low in normal mature tissues, with one exception: HMGA2 is strongly expressed during spermatogenesis in the testis in mice [38]. Intriguingly, independent studies have shown that HMGA2 is re-expressed during oncogenesis in a variety of human malignancies [26, 27], including gastric cancer, where high expression of HMGA2 correlates with lymph node metastasis, increased TNM stage and reduced patient survival in GC patients [28, 39, 40]. The aberrant expression of HMGA2 is regulated by diverse mechanisms, including chromosomal rearrangements [41] or several noncoding RNAs [25]. A recent study also demonstrated that N6-methyladenosine modification of circNSUN2 stabilized HMGA2 mRNA by IGF2BP2 and promoted the aggressiveness of CRC cells [42]. In our study, a high level of HMGA2 in GC was observed in both TCGA dataset and our own samples; more importantly, poorer survival of GC patients with high HMGA2 was also validated. These data suggested the vital role of HMGA2 in GC, which was then confirmed by subsequent functional experiments both in vitro and in vivo. Here, we also elucidated a novel pattern of HMGA2 regulation in which HMGA2 was verified as a direct downstream target of miR-490-3p in GC, while circFAM73A functions as a sponge of miR-490-3p, thus modulating HMGA2 expression. We also proved that the facilitating effects of circFAM73A on GC are, at least partially, mediated by HMGA2.

The biogenesis of circRNAs is intricately involved in multiple factors. Generally, the endogenous level of circRNAs in cells is regulated at three levels, including the transcription of circRNA-producing pre-mRNA and co- or post-transcriptional processing affecting the efficiency of backsplicing and circRNA turnover. It was reported that transcription factors could modulate the biogenesis of circRNAs. CircHIPK3 was enriched in diabetes mellitus and colorectal cancer by c-Myb [43, 44]. Twist1 could bind the Cul2 promoter and activate its transcription, resulting in the selective upregulation of Cul2 circular RNA but not mRNA [45]. In our work, we demonstrated that HMGA2 interacts with pRB and enhances the activity of E2F1, which was consistent with a previous study [26]. E2F1 is a well-known transcription factor with frequent hyperactivation in human malignancy and induces transcription of a number of genes that contribute to cancerous progression [26, 46, 47]. More intriguingly, we validated that E2F1 could bind the FAM73A promoter and activate its transcription, thus elevating the expression of pre-FAM73A, as well as circFAM73A. Therefore, HMGA2 regulated circFAM73A expression by E2F1 activation.

However, elevated pre-FAM73A caused by HMGA2 induction resulted in an increase in circFAM73A but not linear FAM73A mRNA. The uncorrelated levels of circular RNA and linear mRNA prompted us to investigate the possible involvement of HMGA2 in the formation efficiency of pre-FAM73A. Existing studies have established that the binding of *trans* factors in flanking introns could elevate circRNA formation by drawing backsplicing exon ends into close proximity [30, 32]. The processing of circRNAs could modify the alternative splicing of such pre-mRNAs, therefore shifting the output of protein-coding genes to circular RNAs [29, 48]. Even if these effects might be limited, as circular RNAs are naturally resistant to exonucleases, slight alteration of the efficiency of backsplicing might result in profound variations in the steady-state levels of these transcripts. HNRNPL was previously confirmed to promote circRNA formation in prostate cancer [32], and the binding of HNRNPL within the flanking introns of circFAM73A was also verified via motif scanning and RIP assays, strongly indicating its role in circFAM73A formation. Notably, knockdown of HNRNPL not only resulted in a reduction in circFAM73A but also caused a slight increase in FAM73A. A recent study also reported that interfering with SMN circRNA biogenesis leads to the significant rescue of SMN protein [49], indicating competition between canonical splicing and backsplicing, when backsplicing of exons for circRNA formation is considered an unusual type of alternative splicing. This competition might explain the increase in linear FAM73A mRNA caused by HNRNPL depletion, as it impedes the backsplicing of pre-FAM73A. Subsequent investigations verified that HMGA2 increased the backsplicing efficiency in pre-FAM73A by HNRNPL. Nevertheless, further investigations are needed to elucidate the precise mechanism of HMGA2 regulation on HNRNPL.

These observations suggested that HMGA2 could in turn elevate circFAM73A expression by enhancing the transcription of FAM73A by E2F1 and increasing the efficiency of circFAM73A circularization by HNRNPL, which may partially account for the positive correlation between HMGA2 and circFAM73A. These findings reveal a novel feedback loop in circRNA regulation that promotes GC development.

In addition to acting as sponges of miRNAs, circRNAs can also interact with different proteins that subsequently influence the function of associated proteins. To fully explore the mechanisms underlying circFAM73A, we conducted RNA pulldown followed by mass spectrometry analysis to assess the potential associated proteins. HNRNPK, one of the major pre-mRNA-binding proteins, was screened out and verified. The hnRNP family is known to regulate gene expression through the RNA-binding domain and is involved in multiple physiological and pathological processes, such as organogenesis, erythroid differentiation, and carcinogenesis [50, 51]. Increasing evidence suggests that HNRNPK is a key interactor of ncRNAs associated with a variety of aspects of human health and disease [52, 53]. Here, we demonstrated that circFAM73A binds to HNRNPK, enhances the interaction between HNRNPK and β-catenin and facilitates the stability of β-catenin, thus promoting CSC-like properties in gastric cancer.

### Conclusions

In conclusion, our work underscores a novel pathway of circFAM73A that functions in facilitating cancer stem cell-like properties in gastric cancer. More importantly, the upregulation of circFAM73A is closely correlated with the poor prognosis of GC patients. CircFAM73A regulates HMGA2 expression by absorbing miR-490-3p in a positive feedback loop. CircFAM73A also interacts with HNRNPK and facilitates β-catenin stabilization. These findings helped elucidate the progression of gastric cancer and may provide new insight into circRNA-based diagnostic and therapeutic strategies.

## Supplementary Information

**Additional file 1 Table S1**. Primers used in this study.

**Additional file 2 Table S2**. Antibodies used in this study.

**Additional file 3: Supplementary Figure 1**. **(A).** Relative levels of circFAM73A and FAM73A mRNA were measured by qRT-PCR in SGC7901 treated with Actinomycin D for different periods of time. **(B)** Relative levels of GAPDH (positive control for cytoplasmic fraction), U6 (positive control for nuclear fraction), circFAM73A, and FAM73A mRNA from nuclear and cytoplasmic fractions in SGC7901. **(C)** The subcellular localization of circFAM73A in SGC7901 was determined by FISH. DAPI was used for nuclei staining. Scale bar: 20 μm. **(D)** The log10 fold changes of circFAM73A in each paired GC sample were displayed from high to low. **(E)** Proportion of upregulation and downregulation in 120 paired GC samples. **(F)** The association of circFAM73A expression and age through qRT-PCR. **(G)** The association of circFAM73A expression and gender through qRT-PCR. **(H)** The association of circFAM73A expression and tumor site through qRT-PCR. **(I)** The association of circFAM73A expression and lymph node metastasis through qRT-PCR. **(J)** The association of circFAM73A expression and blood vessel invasion through qRT-PCR. **(K)** Relative linear FAM73A mRNA expression in 100 paired GC tissues. **(L)** Overall survival analysis based on FAM73A expression in 100 GC patients. **(M)** Overall survival analysis based on circFAM73A expression in TCGA database. Graph represents mean ± SD; *p < 0.05, **p < 0.01, ***p < 0.001.

**Additional file 4: Supplementary Figure 2**. **(A)** Two small interfering RNAs (siRNAs) specifically targeting the back-splice junction sequences of circFAM73A were designed. **(B)** The efficiencies of two siRNAs were verified by qRT-PCR. **(C)** The efficiency of circFAM73A overexpression vectors was verified by qRT-PCR. **(D)** Quantification of EdU positive cells in BGC823 and SGC7901 transfected with control, circFAM73A siRNA or circFAM73A plasmid. **(E)** Quantification of diameter of organoids transfected with control, circFAM73A siRNA or circFAM73A plasmid. **(F)** Western blot of cyclin proteins related to G1/S transition, including cyclin D1, cyclin E1, and CDK2 after circFAM73A alternation in BGC823 and SGC7901. **(G)** The histogram showed the quantitative analysis of the bands.

**Additional file 5: Supplementary Figure 3**. **(A)** Relative expression of circFAM73A in CDDP-resistant BGC823 and SGC7901 cells and their parental CDDP-sensitive cells. **(B-E)** Cell viability of BGC823 **(B)** or SGC7901 **(C)** cells with or without circFAM73A reconstitution and BGC823CDDP **(D)** or SGC7901CDDP **(E)** cells with or without circFAM73A inhibition was assessed via CCK-8 assays after various concentrations of CDDP stimulation. The IC50 value of each group was also measured. **(F)** Colony formation assays of respective CDDP-sensitive or CDDP-resistant groups after treatment of indicated CDDP concentrations. **(G)** Cell apoptosis were detected by flow cytometry of respective CDDP-sensitive or CDDP-resistant groups after treatment of indicated CDDP concentrations. **(H)** The histogram showed the quantitative analysis of the bands of stemness-related factors including CD44, SOX-2, OCT-4, and Nanog after circFAM73A alternation in BGC823 and SGC7901. Graph represents mean ± SD; *p < 0.05, **p < 0.01, ***p < 0.001.

**Additional file 6: Supplementary Figure 4**. **(A)** Schematic drawing showing the 8 miRNAs that satisfying these criteria. **(B)** The efficiency of circFAM73A probe was confirmed by qRT-PCR in BGC-823 and SGC-7901 cells. **(C)** Relative miR-490-3p expression in 100 paired GC tissues and adjacent tissue. **(D)** Correlation between miR-490-3p and AURKA, ONECUT2, RNF207, and HMGA2 according to TCGA statistics. **(E)** The histogram showed the quantitative analysis of the bands of AURKA, ONECUT2, RNF207, and HMGA2 after miR-490-3p alternation in BGC823. **(F)** The expression of AURKA, ONECUT2, RNF207, and HMGA2 after miR-490-3p alternation in SGC7901 cells measured by qRT-PCR. **(G)** The expression of AURKA, ONECUT2, RNF207, and HMGA2 after miR-490-3p alternation in SGC7901 cells measured by Western blot. The histogram in the right plot showed the quantitative analysis of the bands. **(H)** Cell viability was detected by CCK-8 in BGC823 and SGC7901 cells with or without suppression of AURKA, ONECUT2, RNF207, and HMGA2. **(I)** The flow chart of identification of miR-490-3p target gene. **(J)** Dual-luciferase reporter assays with wild-type or mutant-type HMGA2 3’-UTRs were performed with or without exogenous expression of miR-490-3p in BGC823 cells and SGC7901. Relative fluorescence intensity was quantified. Graph represents mean ± SD; *p < 0.05, **p < 0.01, ***p < 0.001.

**Additional file 7: Supplementary Figure 5**. BGC823 and SGC7901 cells transfected with miR-490-3p mimic or negative control were further transfected with HMGA overexpression plasmids. miR-490-3p suppression or control cells were further constructed with HMGA2 inhibition. **(A, B)** Representative images and quantification of clone formation. **(C, D)** Representative images and quantification of formatted spheres among indicated cells. Scale bar: 100 μm. **(E, F)** Representative images and quantification of migrated cells were tested by Transwell assay among indicated cells. Scale bar: 100 μm. Graph represents mean ± SD; * vs the group of first column, ^#^ vs the group of third column. *p < 0.05, **p < 0.01, ***p < 0.001, ^#^*p* < 0.05, ^# #^*p* < 0.01, ^# # #^*p* < 0.001.

**Additional file 8: Supplementary Figure 6**. **(A)** Relative mRNA levels of HMGA2 were detected by qRT-PCR among indicated cells. **(B)** Western blot of HMGA2 was detected in SGC7901 transfected with indicated vectors. **(C)** The histogram showed the quantitative analysis of the bands of HMGA2 in BGC823 and SGC7901 transfected with indicated vectors. **(D)** Representative flow cytometric histograms and quantification of the CD44 positive proportion in SGC7901 transfected with indicated vectors. **(E)** The histogram showed the quantitative analysis of the bands of CD44, SOX-2, OCT-4, and Nanog in BGC823 transfected with indicated vectors. **(F)** Western blot of stemness-related factors including CD44, SOX-2, OCT-4, and Nanog in SGC7901 transfected with indicated vectors. **(E)** The histogram showed the quantitative analysis of the bands of CD44, SOX-2, OCT-4, and Nanog in SGC7901 transfected with indicated vectors. Graph represents mean ± SD; * vs the group of first column, ^#^ vs the group of third column. *p < 0.05, **p < 0.01, ***p < 0.001, ^#^*p* < 0.05, ^# #^*p* < 0.01, ^# # #^*p* < 0.001.

**Additional file 9: Supplementary Figure 7**. BGC823 cells transfected with empty vector or cirFAM73A overexpression plasmids were further transfected with HMGA knocking-down. cirFAM73A suppression or control BGC823 cells were further reconstructed with HMGA2. **(A)** Representative images and quantification of formatted spheres among indicated cells. Scale bar: 100 μm. **(B)** Representative images and quantification of clone formation among indicated cells. **(C)** Representative images of EdU staining and quantification of EdU positive cells among indicated cells. Scale bar: 100 μm. **(D)** Representative images of cell cycle distribution among indicated cells detected by flow cytometry. **(E)** Representative images and quantification of migrated cells among indicated cells tested by Transwell assay. Scale bar: 100 μm. Graph represents mean ± SD; *p < 0.05, **p < 0.01, ***p < 0.001.

**Additional file 10: Supplementary Figure 8**. **(A, B)** Weight of extracted xenograft tumors after mice were sacrificed. **(C, D)** mRNA levels of HMGA2 and CD44 in respective xenograft tumors samples. * vs the group of first column, ^#^ vs the group of second column. **(E, F)** Metastatic foci in mice livers of each group were counted. Graph represents mean ± SD; *p < 0.05, **p < 0.01, ***p < 0.001, ^#^*p* < 0.05, ^# #^*p* < 0.01, ^# # #^*p* < 0.001.

**Additional file 11: Supplementary Figure 9**. **(A)** qRT-PCR analysis of E2F1 and classical E2F1 responsive effectors in BGC823 and SGC7901. Pseudocolors represent the intensity scale of expression in HMGA2 vs. vector cells and sh-HMGA2 vs. control cells calculated by log2 transformation. **(B)** Western blot of E2F1 and classical E2F1 responsive effectors (CCNE1, TK1, and CDC2) in SGC7901. (**C**) The histogram showed the quantitative analysis of the bands of CCNE1, TK1, and CDC2 after HMGA2 alternation in BGC823 and SGC7901. **(D)** Schematic diagram illustrated the design of specific primers of pre-FAM73A, FAM73A mRNA and circFAM73A. **(E)** Relative expression of pre-FAM73A, FAM73A mRNA and circFAM73A with E2F1 alteration were measured by qRT-PCR in SGC7901. **(F)** Luciferase reporter assay analysis of FAM73A promoter luciferase reporters in SGC7901 cells transfected with E2F1 or control. **(G)** RT-PCR was performed in SGC7901 cells after chromatin immunoprecipitation by E2F1 antibody or control IgG and by two pairs of primers to validate the E2F1 binding sites in FAM73A promoter region. **(H)** qRT-PCR analysis of chromatin immunoprecipitation assay in G. **(I)** Relative expression of pre-FAM73A, FAM73A mRNA and circFAM73A in SGC7901 with HMGA2 reconstitution were measured by qRT-PCR. **(J)** Predicted binding site of HNRNPL in flanking intron-2 and intron-7 of circFAM73A by MEME Suite. **(K)** Relative enrichment of amplification sequence by three indicated primes after RNA binding protein immunoprecipitation assay by HNRNPL antibody or control IgG in SGC7901. **(L)** Relative expression of pre-FAM73A, FAM73A mRNA and circFAM73A in SGC7901 with HNRNPL suppression were measured by qRT-PCR. **(M)** The ratio of circFAM73A expression to FAM73A mRNA expression in SGC7901. Graph represents mean ± SD; *p < 0.05, **p < 0.01, ***p < 0.001.

**Additional file 12: Supplementary Figure 10**. **(A)** Relative abundance of circFAM73A detected by qRT-PCR after RIP using HNRNPK antibody in SGC7901. **(B)** Expression of HNRNPK protein of SGC7901after circFAM73A overexpression or knocking-down measured by Western Blot. The histogram in the right plot showed the quantitative analysis of the bands. **(C)** Co-IP analysis using HNRNPK protein revealing the endogenous interaction between HNRNPK and β-catenin in SGC7901. **(D)** Expression of β-catenin protein in SGC7901after circFAM73A overexpression or knocking-down measured by Western Blot. The histogram in the right plot showed the quantitative analysis of the bands. **(E)** Expression of β-catenin protein in SGC7901transfected with control shRNA or sh-circFAM73A and treated with MG132 (10 μmol/L, 10 h) or untreated measured by Western Blot. The histogram in the right plot showed the quantitative analysis of the bands. **(F)** Expression of β-catenin protein in SGC7901transfected with control shRNA or sh-circFAM73A and treated with cycloheximide (CHX, 50 μg/mL) for different time measured by Western Blot. The graph in the right plot showed the relative intensity of β-catenin at different time points. **(G)** Co-IP and Western blot showing the interaction between HNRNPK and β-catenin after circFAM73A overexpression or knocking-down in SGC7901. The histogram in the right plot showed the quantitative analysis of the bands. **(H)** The nuclear and cytoplasmic expression of HNRNPK and β-catenin measured by Western Blot after circFAM73A overexpression or knocking-down in SGC7901. The histogram in the right plot showed the quantitative analysis of the bands. **(I-K)** Representative images and quantification of clone formation **(I)**, migrated cells **(J)**, and formatted spheres **(K)** in SGC7901transfected with vector or circFAM73A plasmid and co-transfected with control siRNA or si-HNRNPK. Graph represents mean ± SD; *p < 0.05, **p < 0.01, ***p < 0.001.

## Data Availability

All data in this current study are available from the corresponding author upon reasonable request.

## Abbreviations

CSC: Cancer stem cells
circRNA: Circular RNA
GC: Gastric cancer
FISH: Fluorescence in situ hybridization
HMGA2: High Mobility Group A2
IHC: Immunohistochemistry
Pre-mRNA: Precursor mRNA
qRT-PCR: Quantitative real-time polymerase chain reaction
