# Supplementary material for: CircFAM73A promotes the cancer stem cell-like properties of gastric cancer through the miR-490-3p/HMGA2 positive feedback loop and HNRNPK-mediated β-catenin stabilization
Source: J Exp Clin Cancer Res. 2021 Mar 17;40:103. doi: 10.1186/s13046-021-01896-9 (PMC7972245; doi:10.1186/s13046-021-01896-9)
Supplement: Supplementary file 1 — Additional file 1 Table S1. Primers used in this study. [file 13046_2021_1896_MOESM1_ESM.docx]

**Table S1. Primers used in this study.**

| hsa_circ_0001789 | Forward | GGCCTCGGATGGTGGTATAAG |
| --- | --- | --- |
|  | Reverse | GGTCAAACATGCTGGCAGTC |
| hsa_circ_0007376 | Forward | CTCCTACATGGCTGTTCTCCG |
|  | Reverse | TCTAGAGTTCACGAGGATGTTGG |
| hsa_circ_0052001 | Forward | AGCATGCTGCAGGACGTA |
|  | Reverse | ATCTCCACAATGGCTTCTGTGT |
| Divergent-circFAM73A | Forward | ATAAACTCGAAGCTCTGCTGC |
|  | Reverse | TCACTGCAGTTACCACAAACAAC |
| Convergent-circFAM73A | Forward | GGTTCTGCACAGAGTTTGGC |
|  | Reverse | TTCATCTGCTTTGTCCCAGGAA |
| FAM73A mRNA | Forward | ACAGCAGCGCTAAGAGTGTT |
|  | Reverse | TGAGGTGCTCTGGTTCCCAT |
| FAM73A pre-mRNA | Forward | AGGTCCTTCTTTTAAGTGAACAAAT |
|  | Reverse | ATAGCACTCACTGCAGTTACCA |
| GAPDH | Forward | CAATGACCCCTTCATTGACC |
|  | Reverse | TTGATTTTGGAGGGATCTCG |
| Divergent GAPDH | Forward | GAAGGTGAAGGTCGAGTC |
|  | Reverse | GAAGATGGTGATGGGATTTC |
| U6 | Forward | CTCGCTTCGGCAGCACA |
|  | Reverse | AACGCTTCACGAATTTGCGT |
| CD44 | Forward | CTGCCGCTTTGCAGGTGTA |
|  | Reverse | CATTGTGGGCAAGGTGCTATT |
| SOX-2 | Forward | CATCACCCACAGCAAATGACA |
|  | Reverse | GCTCCTACCGTACCACTAGAACTT |
| OCT-4 | Forward | GCAGCGACTATGCACAACGA |
|  | Reverse | CCAGAGTGGTGACGGAGACA |
| Nanog | Forward | TTTGTGGGCCTGAAGAAAACT |
|  | Reverse | AGGGCTGTCCTGAATAAGCAG |
| AURKA | Forward | GAGGTCCAAAACGTGTTCTCG |
|  | Reverse | ACAGGATGAGGTACACTGGTTG |
| [ONECUT2](https://www.ncbi.nlm.nih.gov/gene/9480) | Forward | AACGCAAAGAGCAAGAACCAA |
|  | Reverse | AAGATGGCGAAGAGTGTTCGG |
| [RNF207](https://www.ncbi.nlm.nih.gov/gene/388591) | Forward | GGCGGAGATCATGGGAGAC |
|  | Reverse | CACTCGCTGATAGGCTTCCTC |
| HMGA2 | Forward | ACCCAGGGGAAGACCCAAA |
|  | Reverse | CCTCTTGGCCGTTTTTCTCCA |
| CDC2 | Forward | AACTACAGGTCAAGTGGTAG |
|  | Reverse | TACTCTGATAGGTATTCCAA |
| CCNE1 | Forward | GAGCTTGTTCAGGAGATGAA |
|  | Reverse | ACACAGAGATCCAACAGCTC |
| TK1 | Forward | CTGGTGATCAAGTATGCCAA |
|  | Reverse | GCCTTCTTGAAGTAGCAGAG |
| E2F1 | Forward | AGCGGCGCATCTATGACATC |
|  | Reverse | TCCTGGGTCAACCCCTCAA |
| ChIP Primer 1 | Forward | ACCGGTTTGTAAAAGGATGCAA |
|  | Reverse | TTTCTGCGTGAGGGACTACG |
| ChIP Primer 2 | Forward | GGCTGGTCTTGAACTCCTGA |
|  | Reverse | AACAACAACAAAACCCAAGG |
| RIP Primer 1 | Forward | AGGCTCTGGTGAGCTGTGAT |
|  | Reverse | GTGGAAAAGTGTGGCAGACA |
| RIP Primer 2 | Forward | CAAAGGGCCCACCTAGTACA |
|  | Reverse | AAAATGCTGGGTTTGCAGTC |
| RIP Primer 3 | Forward | TTTGAAGTTTGCTAAAAGGCTAGAA |
|  | Reverse | GCAATGTGAAATGACCACCA |
| hsa-miR-26a-5p | Forward | TATGGAAAGACTTTGTTACTCT |
| hsa-miR-9-5p | Forward | TACTCCAGAGGGCGTCACTCATG |
| hsa-miR-490-3p | Forward | GTGGCTGCACTCACTTCCTTC |
| hsa-miR-326 | Forward | CCTCCCACACCCAAGGCTTGCA |
| hsa-miR-5683 | Forward | GCCCTGAACGAGGGGTCTGGAG |
| hsa-miR-378c | Forward | TCTCTGGTCTTGCCACCCCAG |
| hsa-miR-378d | Forward | TCTGGTCCTGGACAGGAGGC |
| hsa-miR-383-5p | Forward | GGTCCAGAGGGGAGATAGGTTC |
| Universal 5’ primer |  | GCGAGCACAGAATTAATACGAC |
