# Supplementary material for: CircFAM73A promotes the cancer stem cell-like properties of gastric cancer through the miR-490-3p/HMGA2 positive feedback loop and HNRNPK-mediated β-catenin stabilization
Source: J Exp Clin Cancer Res. 2021 Mar 17;40:103. doi: 10.1186/s13046-021-01896-9 (PMC7972245; doi:10.1186/s13046-021-01896-9)
Supplement: Supplementary file 2 — Additional file 2 Table S2. Antibodies used in this study. [file 13046_2021_1896_MOESM2_ESM.docx]

**Table S2. Antibodies used in this study.**

| Antibody | Supplier | Catalogue | Host |
| --- | --- | --- | --- |
| HMGA2 | Proteintech | 20795-1-AP | Rabbit |
| CD44 | Abcam | ab157107 | Rabbit |
| SOX-2 | Proteintech | 11064-1-AP | Rabbit |
| OCT-4 | Proteintech | 11263-1-AP | Rabbit |
| Nanog | Proteintech | 67255-1-Ig | Rabbit |
| AURKA | CST | 91590 | Rabbit |
| ONECUT2 | Abcam | ab28466 | Rabbit |
| RNF207 | Abcam | ab181104 | Rabbit |
| pRB | CST | 8516 | Rabbit |
| CDC2 | CST | 9116 | Mouse |
| CCNE1 | Proteintech | 11554-1-AP | Rabbit |
| TK1 | Proteintech | 15691-1-AP | Rabbit |
| E2F1 | CST | 3742 | Rabbit |
| HNRNPL | CST | 65043 | Rabbit |
| HNRNPK | Abcam | ab52600 | Rabbit |
| Beta-catenin | Abcam | ab16051 | Rabbit |
| Histone H3 | Abcam | ab1791 | Rabbit |
| GAPDH | Abcam | ab8245 | Mouse |
| FITC-CD44 | BD Biosciences | 555478 | Mouse |
| FITC Mouse IgG2b κ Isotype Control | BD Biosciences | 556655 | Mouse |
