# Supplementary figures and images for: CircFAM73A promotes the cancer stem cell-like properties of gastric cancer through the miR-490-3p/HMGA2 positive feedback loop and HNRNPK-mediated β-catenin stabilization
Source: J Exp Clin Cancer Res. 2021 Mar 17;40:103. doi: 10.1186/s13046-021-01896-9 (PMC7972245; doi:10.1186/s13046-021-01896-9)

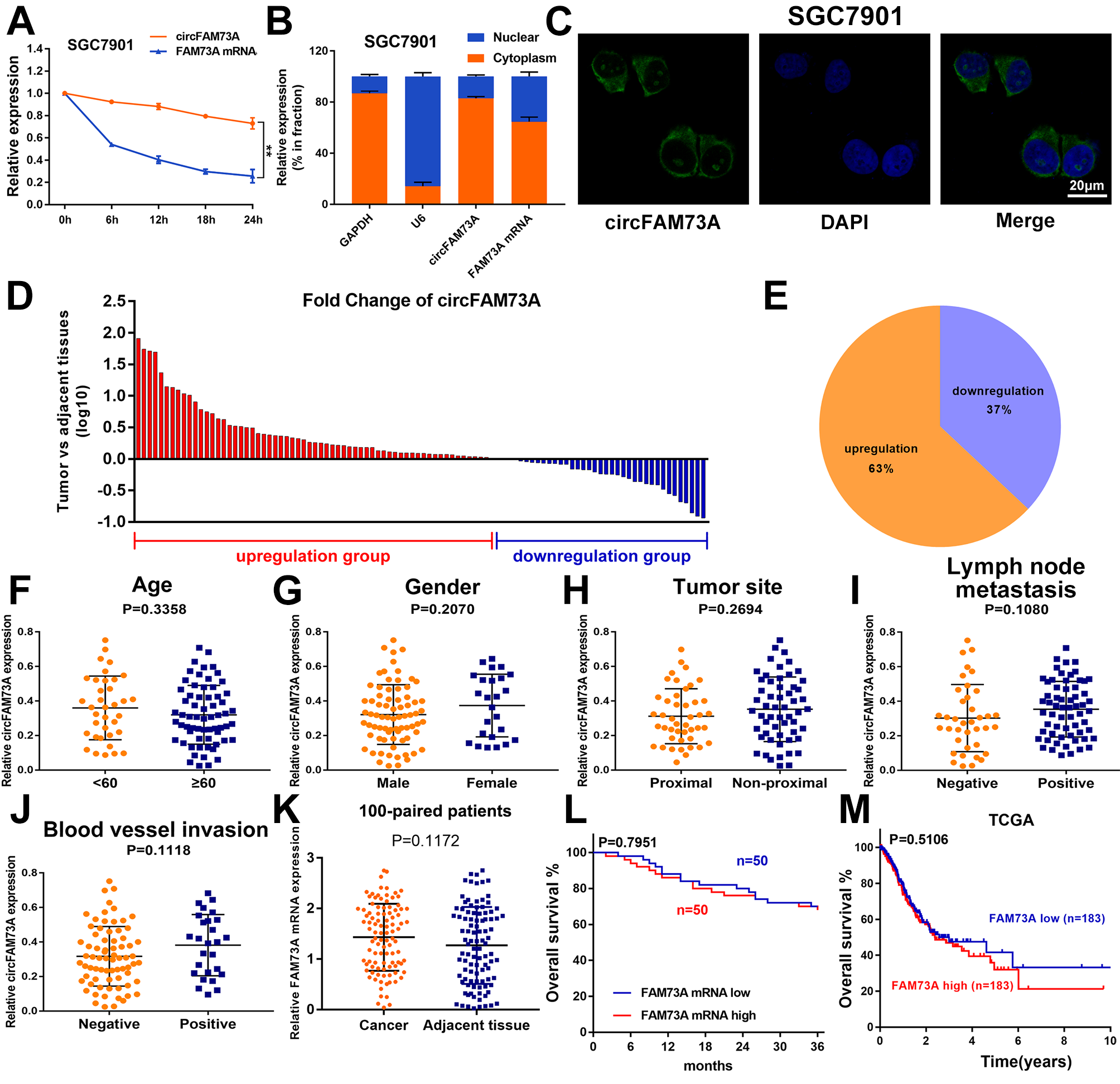

Supplement: Supplementary file 3 — Additional file 3: Supplementary Figure 1. (A). Relative levels of circFAM73A and FAM73A mRNA were measured by qRT-PCR in SGC7901 treated with Actinomycin D for different periods of time. (B) Relative levels of GAPDH (positive control for cytoplasmic fraction), U6 (positive control for nuclear fraction), circFAM73A, and FAM73A mRNA from nuclear and cytoplasmic fractions in SGC7901. (C) The subcellular localization of circFAM73A in SGC7901 was determined by FISH. DAPI was used for nuclei staining. Scale bar: 20 μm. (D) The log10 fold changes of circFAM73A in each paired GC sample were displayed from high to low. (E) Proportion of upregulation and downregulation in 120 paired GC samples. (F) The association of circFAM73A expression and age through qRT-PCR. (G) The association of circFAM73A expression and gender through qRT-PCR. (H) The association of circFAM73A expression and tumor site through qRT-PCR. (I) The association of circFAM73A expression and lymph node metastasis through qRT-PCR. (J) The association of circFAM73A expression and blood vessel invasion through qRT-PCR. (K) Relative linear FAM73A mRNA expression in 100 paired GC tissues. (L) Overall survival analysis based on FAM73A expression in 100 GC patients. (M) Overall survival analysis based on circFAM73A expression in TCGA database. Graph represents mean ± SD; *p < 0.05, **p < 0.01, ***p < 0.001. [file 13046_2021_1896_MOESM3_ESM.tif]

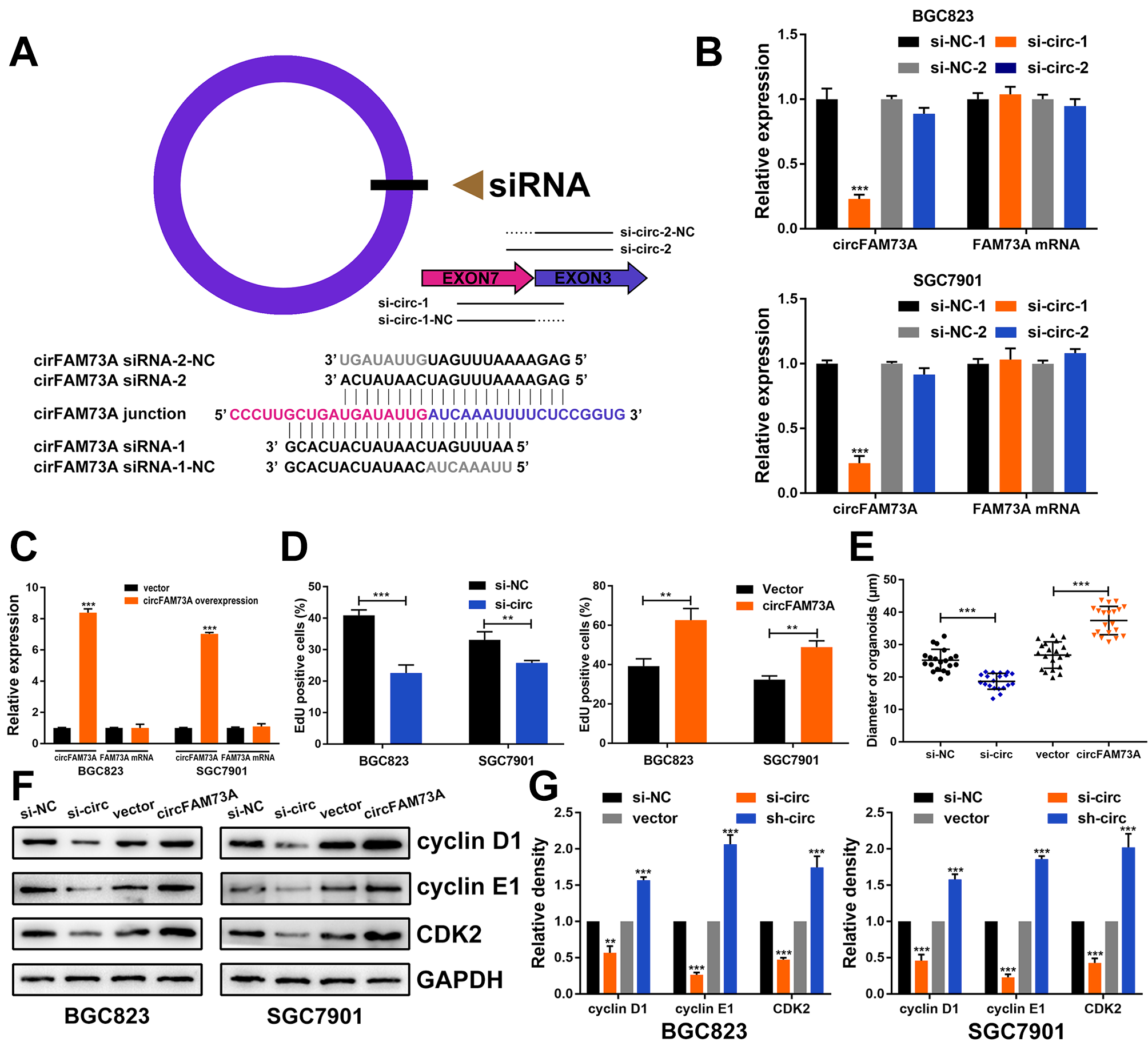

Supplement: Supplementary file 4 — Additional file 4: Supplementary Figure 2. (A) Two small interfering RNAs (siRNAs) specifically targeting the back-splice junction sequences of circFAM73A were designed. (B) The efficiencies of two siRNAs were verified by qRT-PCR. (C) The efficiency of circFAM73A overexpression vectors was verified by qRT-PCR. (D) Quantification of EdU positive cells in BGC823 and SGC7901 transfected with control, circFAM73A siRNA or circFAM73A plasmid. (E) Quantification of diameter of organoids transfected with control, circFAM73A siRNA or circFAM73A plasmid. (F) Western blot of cyclin proteins related to G1/S transition, including cyclin D1, cyclin E1, and CDK2 after circFAM73A alternation in BGC823 and SGC7901. (G) The histogram showed the quantitative analysis of the bands. [file 13046_2021_1896_MOESM4_ESM.tif]

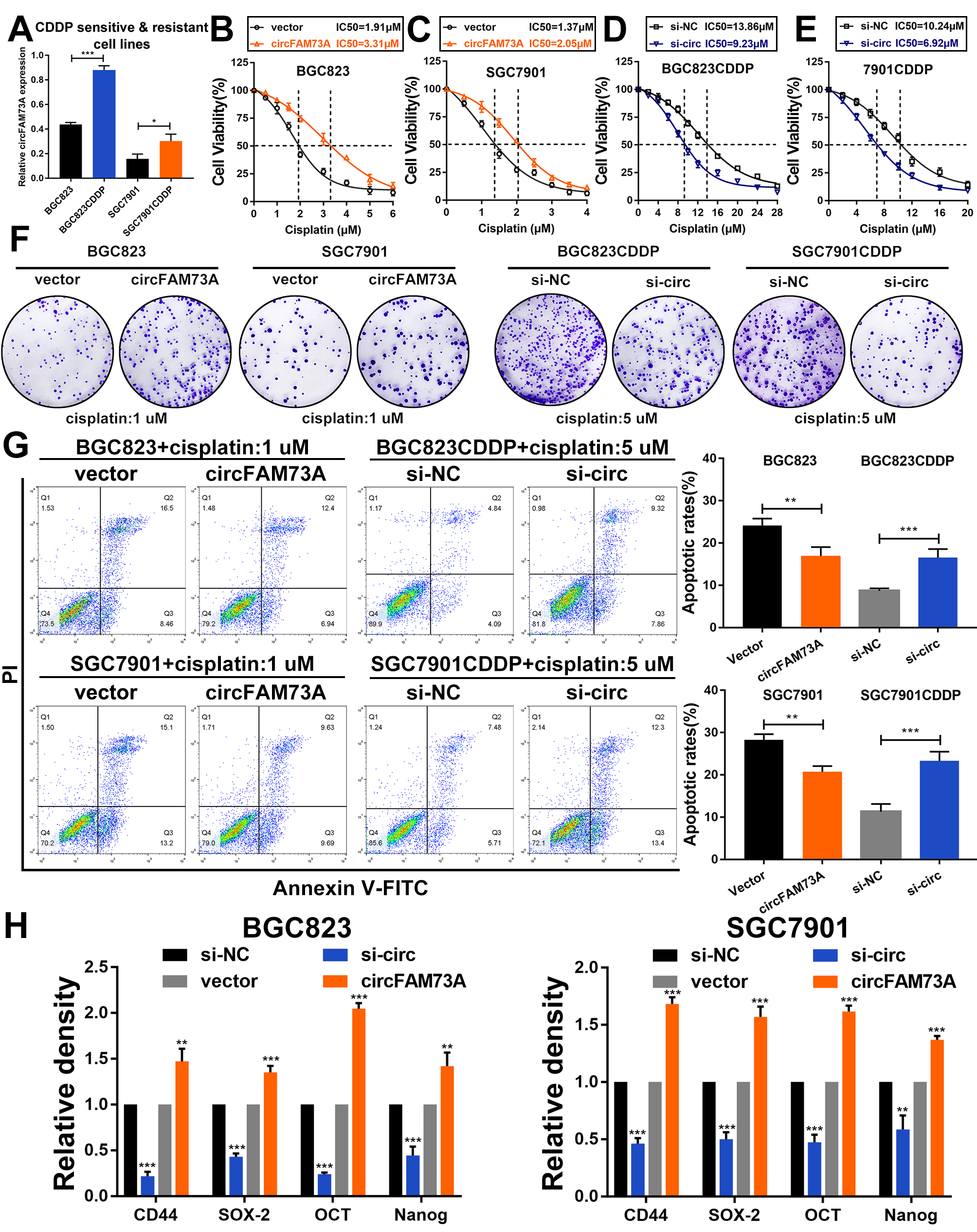

Supplement: Supplementary file 5 — Additional file 5: Supplementary Figure 3. (A) Relative expression of circFAM73A in CDDP-resistant BGC823 and SGC7901 cells and their parental CDDP-sensitive cells. (B-E) Cell viability of BGC823 (B) or SGC7901 (C) cells with or without circFAM73A reconstitution and BGC823CDDP (D) or SGC7901CDDP (E) cells with or without circFAM73A inhibition was assessed via CCK-8 assays after various concentrations of CDDP stimulation. The IC50 value of each group was also measured. (F) Colony formation assays of respective CDDP-sensitive or CDDP-resistant groups after treatment of indicated CDDP concentrations. (G) Cell apoptosis were detected by flow cytometry of respective CDDP-sensitive or CDDP-resistant groups after treatment of indicated CDDP concentrations. (H) The histogram showed the quantitative analysis of the bands of stemness-related factors including CD44, SOX-2, OCT-4, and Nanog after circFAM73A alternation in BGC823 and SGC7901. Graph represents mean ± SD; *p < 0.05, **p < 0.01, ***p < 0.001. [file 13046_2021_1896_MOESM5_ESM.tif]

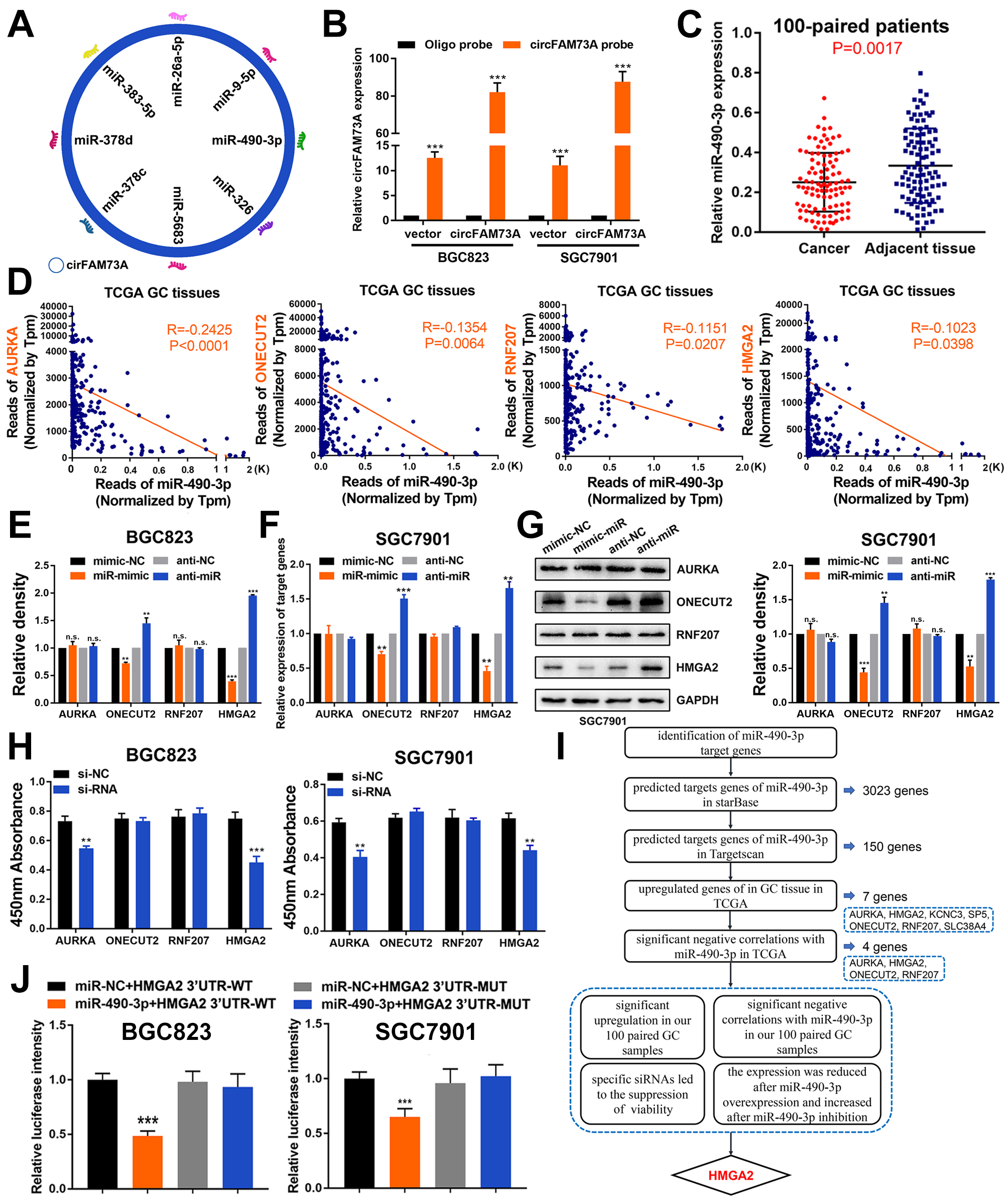

Supplement: Supplementary file 6 — Additional file 6: Supplementary Figure 4. (A) Schematic drawing showing the 8 miRNAs that satisfying these criteria. (B) The efficiency of circFAM73A probe was confirmed by qRT-PCR in BGC-823 and SGC-7901 cells. (C) Relative miR-490-3p expression in 100 paired GC tissues and adjacent tissue. (D) Correlation between miR-490-3p and AURKA, ONECUT2, RNF207, and HMGA2 according to TCGA statistics. (E) The histogram showed the quantitative analysis of the bands of AURKA, ONECUT2, RNF207, and HMGA2 after miR-490-3p alternation in BGC823. (F) The expression of AURKA, ONECUT2, RNF207, and HMGA2 after miR-490-3p alternation in SGC7901 cells measured by qRT-PCR. (G) The expression of AURKA, ONECUT2, RNF207, and HMGA2 after miR-490-3p alternation in SGC7901 cells measured by Western blot. The histogram in the right plot showed the quantitative analysis of the bands. (H) Cell viability was detected by CCK-8 in BGC823 and SGC7901 cells with or without suppression of AURKA, ONECUT2, RNF207, and HMGA2. (I) The flow chart of identification of miR-490-3p target gene. (J) Dual-luciferase reporter assays with wild-type or mutant-type HMGA2 3’-UTRs were performed with or without exogenous expression of miR-490-3p in BGC823 cells and SGC7901. Relative fluorescence intensity was quantified. Graph represents mean ± SD; *p < 0.05, **p < 0.01, ***p < 0.001. [file 13046_2021_1896_MOESM6_ESM.tif]

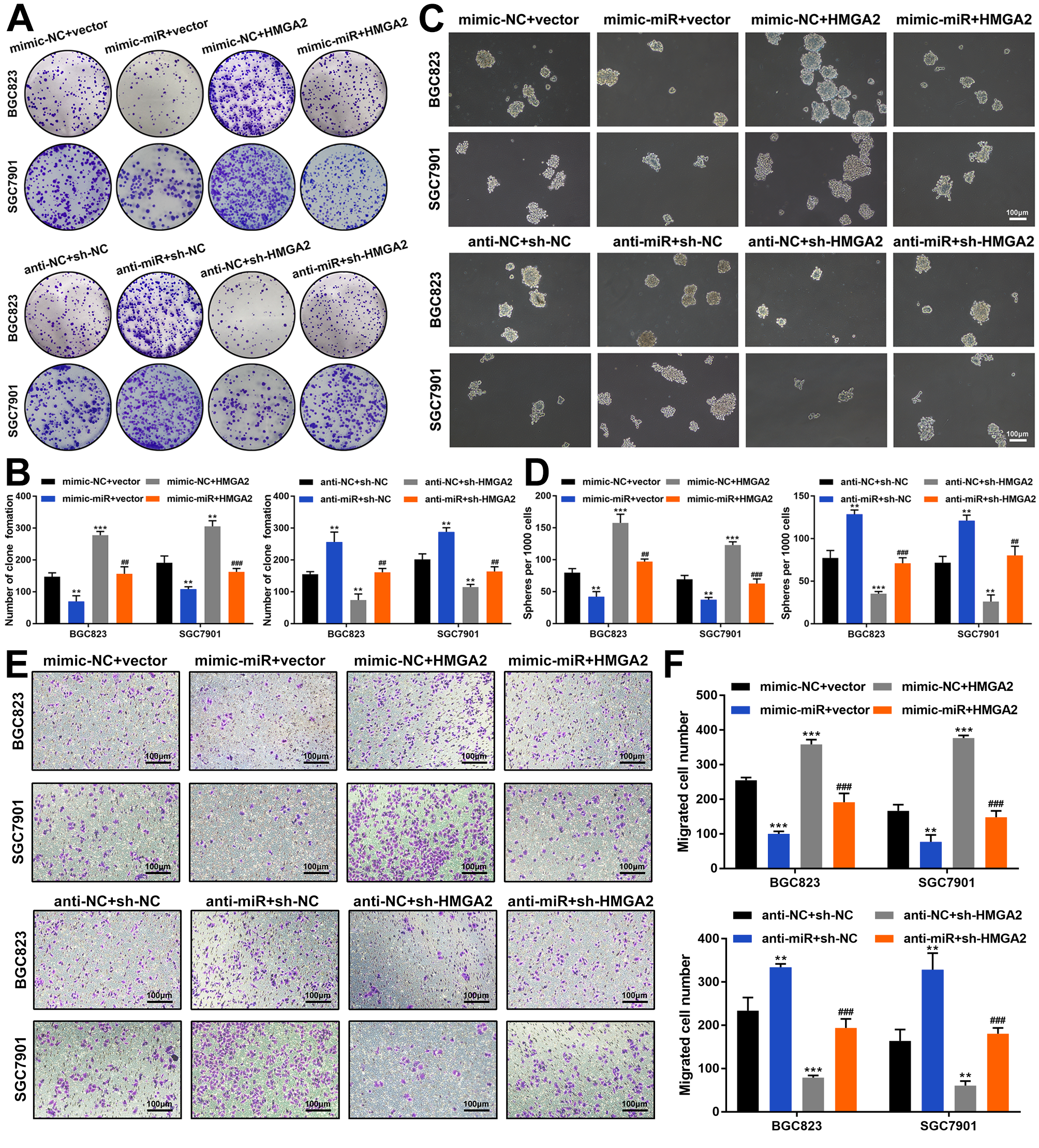

Supplement: Supplementary file 7 — Additional file 7: Supplementary Figure 5. BGC823 and SGC7901 cells transfected with miR-490-3p mimic or negative control were further transfected with HMGA overexpression plasmids. miR-490-3p suppression or control cells were further constructed with HMGA2 inhibition. (A, B) Representative images and quantification of clone formation. (C, D) Representative images and quantification of formatted spheres among indicated cells. Scale bar: 100 μm. (E, F) Representative images and quantification of migrated cells were tested by Transwell assay among indicated cells. Scale bar: 100 μm. Graph represents mean ± SD; * vs the group of first column, # vs the group of third column. *p < 0.05, **p < 0.01, ***p < 0.001, #p < 0.05, # #p < 0.01, # # #p < 0.001. [file 13046_2021_1896_MOESM7_ESM.tif]

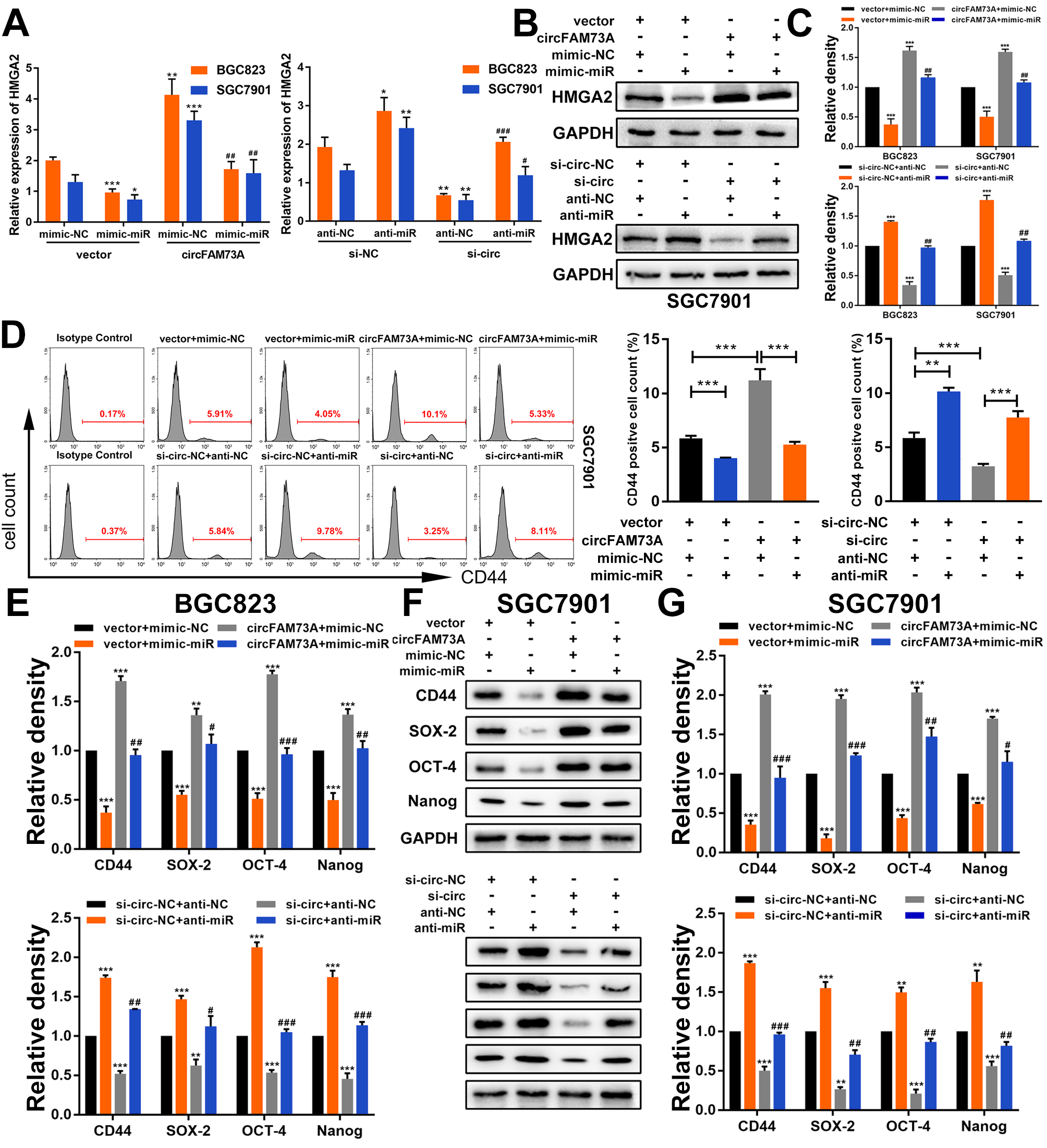

Supplement: Supplementary file 8 — Additional file 8: Supplementary Figure 6. (A) Relative mRNA levels of HMGA2 were detected by qRT-PCR among indicated cells. (B) Western blot of HMGA2 was detected in SGC7901 transfected with indicated vectors. (C) The histogram showed the quantitative analysis of the bands of HMGA2 in BGC823 and SGC7901 transfected with indicated vectors. (D) Representative flow cytometric histograms and quantification of the CD44 positive proportion in SGC7901 transfected with indicated vectors. (E) The histogram showed the quantitative analysis of the bands of CD44, SOX-2, OCT-4, and Nanog in BGC823 transfected with indicated vectors. (F) Western blot of stemness-related factors including CD44, SOX-2, OCT-4, and Nanog in SGC7901 transfected with indicated vectors. (E) The histogram showed the quantitative analysis of the bands of CD44, SOX-2, OCT-4, and Nanog in SGC7901 transfected with indicated vectors. Graph represents mean ± SD; * vs the group of first column, # vs the group of third column. *p < 0.05, **p < 0.01, ***p < 0.001, #p < 0.05, # #p < 0.01, # # #p < 0.001. [file 13046_2021_1896_MOESM8_ESM.tif]

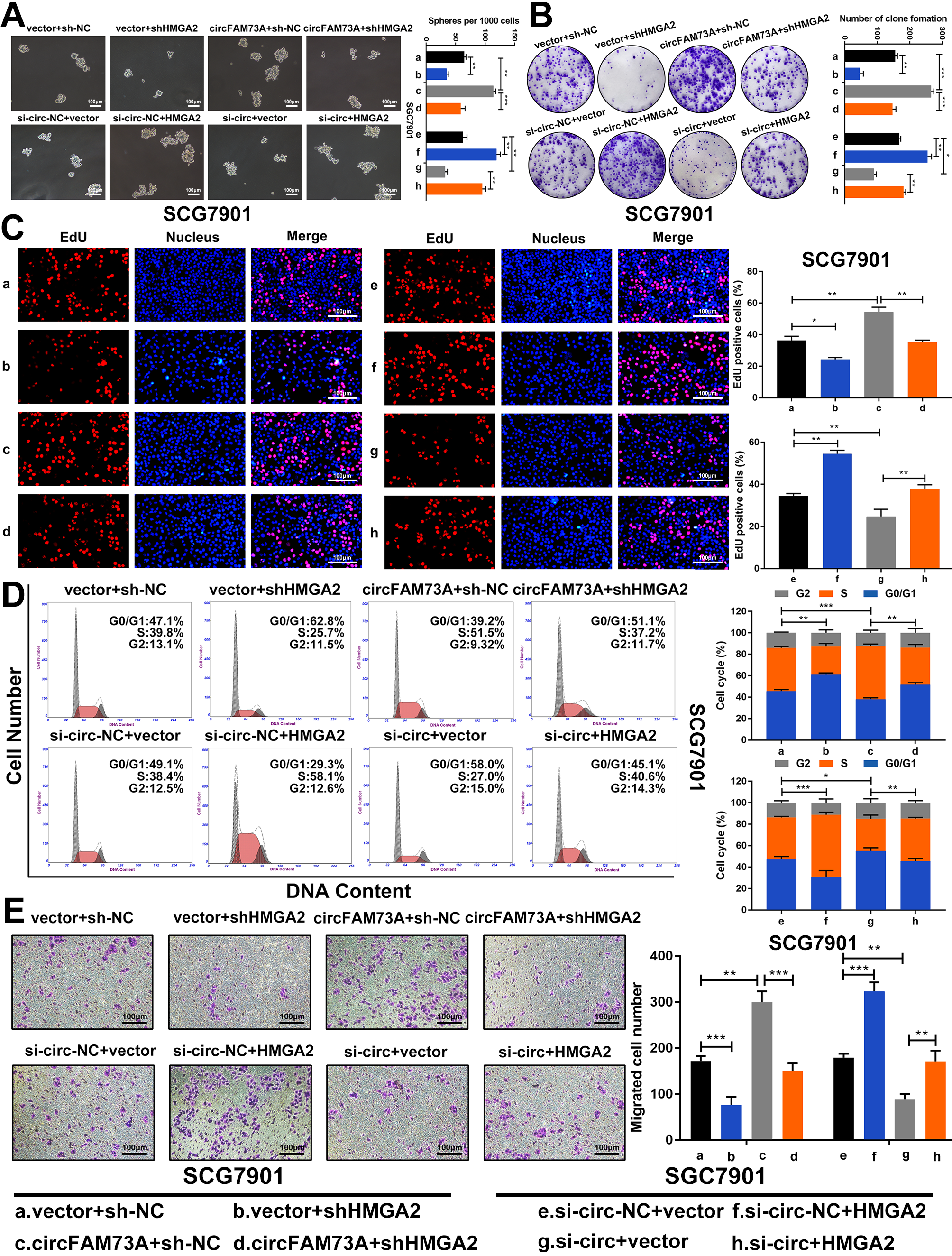

Supplement: Supplementary file 9 — Additional file 9: Supplementary Figure 7. BGC823 cells transfected with empty vector or cirFAM73A overexpression plasmids were further transfected with HMGA knocking-down. cirFAM73A suppression or control BGC823 cells were further reconstructed with HMGA2. (A) Representative images and quantification of formatted spheres among indicated cells. Scale bar: 100 μm. (B) Representative images and quantification of clone formation among indicated cells. (C) Representative images of EdU staining and quantification of EdU positive cells among indicated cells. Scale bar: 100 μm. (D) Representative images of cell cycle distribution among indicated cells detected by flow cytometry. (E) Representative images and quantification of migrated cells among indicated cells tested by Transwell assay. Scale bar: 100 μm. Graph represents mean ± SD; *p < 0.05, **p < 0.01, ***p < 0.001. [file 13046_2021_1896_MOESM9_ESM.tif]

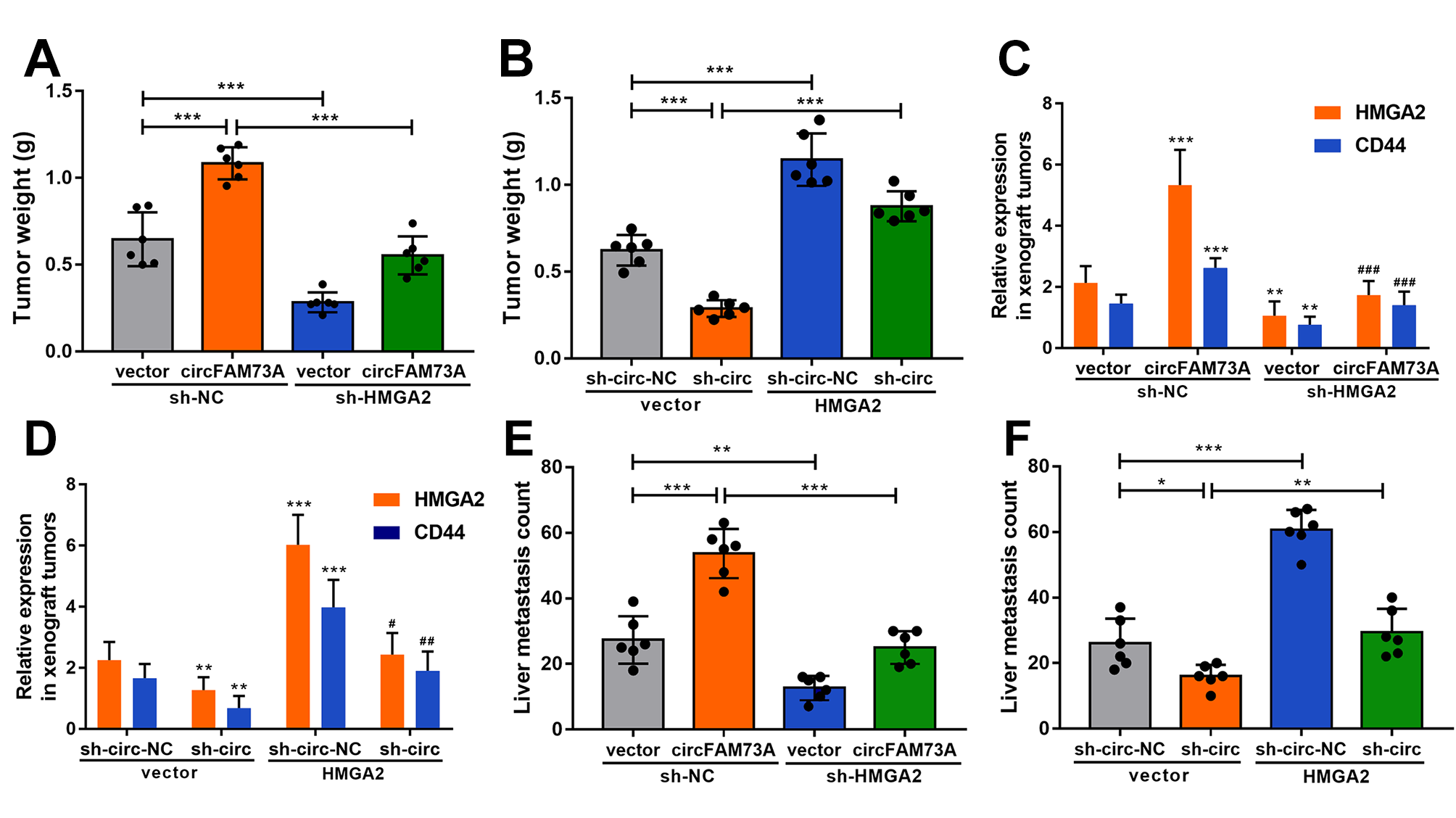

Supplement: Supplementary file 10 — Additional file 10: Supplementary Figure 8. (A, B) Weight of extracted xenograft tumors after mice were sacrificed. (C, D) mRNA levels of HMGA2 and CD44 in respective xenograft tumors samples. * vs the group of first column, # vs the group of second column. (E, F) Metastatic foci in mice livers of each group were counted. Graph represents mean ± SD; *p < 0.05, **p < 0.01, ***p < 0.001, #p < 0.05, # #p < 0.01, # # #p < 0.001. [file 13046_2021_1896_MOESM10_ESM.tif]

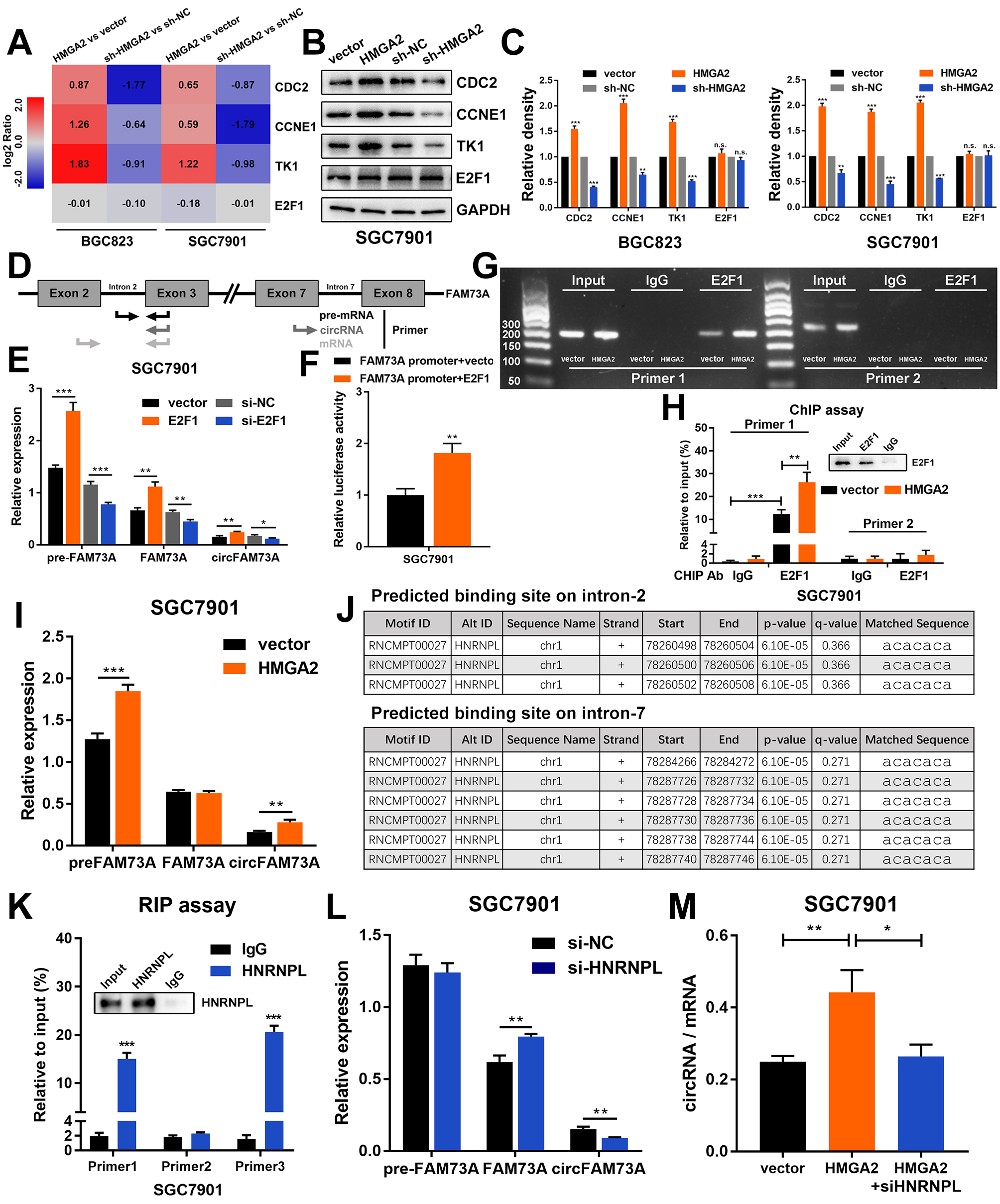

Supplement: Supplementary file 11 — Additional file 11: Supplementary Figure 9. (A) qRT-PCR analysis of E2F1 and classical E2F1 responsive effectors in BGC823 and SGC7901. Pseudocolors represent the intensity scale of expression in HMGA2 vs. vector cells and sh-HMGA2 vs. control cells calculated by log2 transformation. (B) Western blot of E2F1 and classical E2F1 responsive effectors (CCNE1, TK1, and CDC2) in SGC7901. (C) The histogram showed the quantitative analysis of the bands of CCNE1, TK1, and CDC2 after HMGA2 alternation in BGC823 and SGC7901. (D) Schematic diagram illustrated the design of specific primers of pre-FAM73A, FAM73A mRNA and circFAM73A. (E) Relative expression of pre-FAM73A, FAM73A mRNA and circFAM73A with E2F1 alteration were measured by qRT-PCR in SGC7901. (F) Luciferase reporter assay analysis of FAM73A promoter luciferase reporters in SGC7901 cells transfected with E2F1 or control. (G) RT-PCR was performed in SGC7901 cells after chromatin immunoprecipitation by E2F1 antibody or control IgG and by two pairs of primers to validate the E2F1 binding sites in FAM73A promoter region. (H) qRT-PCR analysis of chromatin immunoprecipitation assay in G. (I) Relative expression of pre-FAM73A, FAM73A mRNA and circFAM73A in SGC7901 with HMGA2 reconstitution were measured by qRT-PCR. (J) Predicted binding site of HNRNPL in flanking intron-2 and intron-7 of circFAM73A by MEME Suite. (K) Relative enrichment of amplification sequence by three indicated primes after RNA binding protein immunoprecipitation assay by HNRNPL antibody or control IgG in SGC7901. (L) Relative expression of pre-FAM73A, FAM73A mRNA and circFAM73A in SGC7901 with HNRNPL suppression were measured by qRT-PCR. (M) The ratio of circFAM73A expression to FAM73A mRNA expression in SGC7901. Graph represents mean ± SD; *p < 0.05, **p < 0.01, ***p < 0.001. [file 13046_2021_1896_MOESM11_ESM.tif]

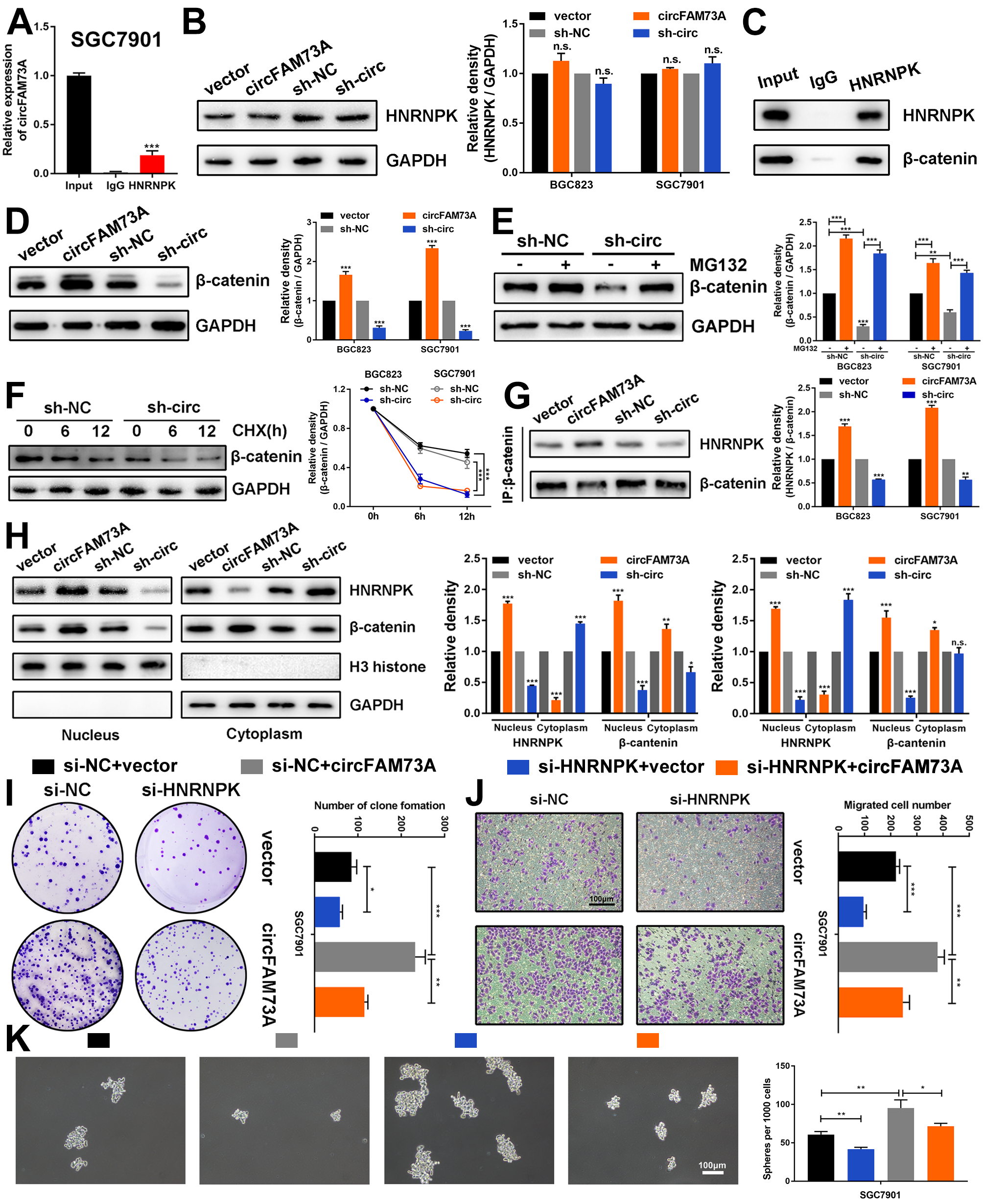

Supplement: Supplementary file 12 — Additional file 12: Supplementary Figure 10. (A) Relative abundance of circFAM73A detected by qRT-PCR after RIP using HNRNPK antibody in SGC7901. (B) Expression of HNRNPK protein of SGC7901after circFAM73A overexpression or knocking-down measured by Western Blot. The histogram in the right plot showed the quantitative analysis of the bands. (C) Co-IP analysis using HNRNPK protein revealing the endogenous interaction between HNRNPK and β-catenin in SGC7901. (D) Expression of β-catenin protein in SGC7901after circFAM73A overexpression or knocking-down measured by Western Blot. The histogram in the right plot showed the quantitative analysis of the bands. (E) Expression of β-catenin protein in SGC7901transfected with control shRNA or sh-circFAM73A and treated with MG132 (10 μmol/L, 10 h) or untreated measured by Western Blot. The histogram in the right plot showed the quantitative analysis of the bands. (F) Expression of β-catenin protein in SGC7901transfected with control shRNA or sh-circFAM73A and treated with cycloheximide (CHX, 50 μg/mL) for different time measured by Western Blot. The graph in the right plot showed the relative intensity of β-catenin at different time points. (G) Co-IP and Western blot showing the interaction between HNRNPK and β-catenin after circFAM73A overexpression or knocking-down in SGC7901. The histogram in the right plot showed the quantitative analysis of the bands. (H) The nuclear and cytoplasmic expression of HNRNPK and β-catenin measured by Western Blot after circFAM73A overexpression or knocking-down in SGC7901. The histogram in the right plot showed the quantitative analysis of the bands. (I-K) Representative images and quantification of clone formation (I), migrated cells (J), and formatted spheres (K) in SGC7901transfected with vector or circFAM73A plasmid and co-transfected with control siRNA or si-HNRNPK. Graph represents mean ± SD; *p < 0.05, **p < 0.01, ***p < 0.001. [file 13046_2021_1896_MOESM12_ESM.tif]
